# A new approach to describe the taxonomic structure of microbiome and its application to assess the relationship between microbial niches

**DOI:** 10.1186/s12859-023-05575-8

**Published:** 2024-02-05

**Authors:** Vincent Y. Pappalardo, Leyla Azarang, Egija Zaura, Bernd W. Brandt, Renée X. de Menezes

**Affiliations:** 1 Department of Preventive Dentistry, Academic Centre for Dentistry Amsterdam, University of Amsterdam and Vrije Universiteit Amsterdam, Amsterdam, The Netherlands; 2 Biostatistics Centre, Department of Psychosocial Research and Epidemiology, Netherlands Cancer Institute, Amsterdam, The Netherlands

**Keywords:** Latent Dirichlet allocation, Oral microbiome, Unsupervised machine learning, Bacterial sub-communities

## Abstract

**Background:**

Data from microbiomes from multiple niches is often collected, but methods to analyse these often ignore associations between niches. One interesting case is that of the oral microbiome. Its composition is receiving increasing attention due to reports on its associations with general health. While the oral cavity includes different niches, multi-niche microbiome data analysis is conducted using a single niche at a time and, therefore, ignores other niches that could act as confounding variables. Understanding the interaction between niches would assist interpretation of the results, and help improve our understanding of multi-niche microbiomes.

**Methods:**

In this study, we used a machine learning technique called latent Dirichlet allocation (LDA) on two microbiome datasets consisting of several niches. LDA was used on both individual niches and all niches simultaneously. On individual niches, LDA was used to decompose each niche into bacterial sub-communities unveiling their taxonomic structure. These sub-communities were then used to assess the relationship between microbial niches using the global test. On all niches simultaneously, LDA allowed us to extract meaningful microbial patterns. Sets of co-occurring operational taxonomic units (OTUs) comprising those patterns were then used to predict the original location of each sample.

**Results:**

Our approach showed that the per-niche sub-communities displayed a strong association between supragingival plaque and saliva, as well as between the anterior and posterior tongue. In addition, the LDA-derived microbial signatures were able to predict the original sample niche illustrating the meaningfulness of our sub-communities. For the multi-niche oral microbiome dataset we had an overall accuracy of 76%, and per-niche sensitivity of up to 83%. Finally, for a second multi-niche microbiome dataset from the entire body, microbial niches from the oral cavity displayed stronger associations to each other than with those from other parts of the body, such as niches within the vagina and the skin.

**Conclusion:**

Our LDA-based approach produces sets of co-occurring taxa that can describe niche composition. LDA-derived microbial signatures can also be instrumental in summarizing microbiome data, for both descriptions as well as prediction.

**Supplementary Information:**

The online version contains supplementary material available at 10.1186/s12859-023-05575-8.

## Introduction

The human microbiome consists of trillions of microbial cells living in our body and is therefore comparable with the number of our own cells [1]. Those microbes are found in various locations like the gut, the skin, or the oral cavity, creating complex ecosystems of bacteria, archaea, fungi, and viruses. The oral cavity is an interesting example to be considered: its composition is highly dependent on biological and environmental factors such as the host’s genetics, diet, and oral hygiene. Its microbial composition also varies among different locations in the oral cavity (such as the tongue, the gingiva, the buccal mucosa, and various surfaces around the teeth). Recently, Next Generation Sequencing techniques have provided us with a plethora of new datasets, including oral microbiome composition from multiple oral niches. By analyzing these datasets, several studies have concluded that oral microbial composition is associated with oral diseases such as periodontitis [2], caries, and oral cancer [3]. Other more distant associations have also been posited, such as depression [4], Alzheimer’s disease [5] or diabetes [6].

These studies were generally performed by studying a single oral niche at a time and were therefore unable to delineate potential interactions between oral niches. The salivary microbiome is for example often used as a proxy for the entire oral cavity. However, interactions between bacteria found in different niches can potentially influence the interpretation of effects found on a single niche. By analysing multiple niches of the microbial environment simultaneously, we can uncover relationships between them, which have so far been little explored.

Analysis of microbiome data poses a challenge: models need to take into account compositionality, sparsity, and over-dispersion, as well as how it is normalized. Classical statistical tools can therefore not be applied [7–10]. Here we will focus on the problem of dimension reduction, so in summarizing a microbial profile using a smaller number of variables which can help interpretation. In particular, we will consider the problem of dimension reduction in a context where multiple microbial niches are evaluated for the same samples.

One way to reduce dimensions is by selecting Operational Taxonomic Units (OTUs) that discriminate niches the most. Current methods include those implemented in R packages edgeR [11], DESeq2 [12] or LEfSe [13]. However, by analyzing the OTUs one by one, these techniques ignore both potential relationships between OTUs, as well as the compositional aspect of the data. Other methods aim to take into account the entire composition of the microbiome, such as PERMANOVA [14], ANOSIM [15] as well as several indices to compare diversity between samples (alpha [16] or beta [17] diversity). While these approaches can discriminate microbial composition both over time and between niches [18], by reducing the microbiome data to a single number they over-simplify the problem, for example by making it harder to understand underlying patterns of differences between microbial niches.

Recently, several papers have proposed to use a Dirichlet-Multinomial distribution to model microbiome data [19–21]. This essentially leads to dimension reduction by collapsing the complete microbiome into a few bacterial sub-communities, each representing a set of OTUs. Each sample is then assigned to a single sub-community, depending on its OTU composition. Another method recently applied to analyze microbiome data is Latent Dirichlet Allocation (LDA) [22, 23]. This is also proposed to collapse the microbiome into sub-communities, where each sample is represented as a mixture of several sub-communities, rather than only one sub-community. LDA-derived sub-communities have been shown to display an association with covariates, such as diet [24], host country [25] and even soil biodiversity [26]. To the best of our knowledge, LDA has so far only been used to analyze microbiome of one niche at a time.

In this paper, we propose to use LDA for dimension reduction of multi-niche microbiome data. Our first research aim is to extract a bacterial signature composed of a set of different Operational Taxonomic Units (OTUs) or bacterial taxa for each niche. Secondly, we plan to describe the microbiome structure within one niche, by decomposing each niche into the OTUs/taxa that naturally co-occurred. These will be grouped into subsets, which we will call sub-communities. The third and last aim is to assess the relationship between different microbial niches.

To achieve these aims, we propose an approach to study data from multiple niches using both the LDA-derived sub-communities and the OTU-based microbiome. The approaches presented here enable researchers to both describe the taxonomic structure of a microbial niche by finding sub-communities that characterize it from others, and to find associations between different niches.

This article is organized as follows. In the Methods section, we describe our proposed LDA-based approach, as well as the datasets we will use. In the Results section we show that our approach can be used to identify signatures of microbial niches. Finally, in the last section we outline some remaining challenges, including how to choose the number of communities.

## Methods

### Latent Dirichlet allocation

#### In the context of text-mining

The Latent Dirichlet Allocation (LDA) is a generative statistical model usually applied in the context of text-mining analysis for the clustering of documents, where each topic is treated as a cluster. For a given list of documents and the frequency of their terms, LDA is able to extract unobserved topics among those documents. The basic assumption is that documents are combinations of latent topics and topics are combinations of terms. In order to detect latent topics in a collection of documents by LDA, we need to introduce a collection of documents as a document-term matrix, which usually is sparse and high-dimensional. A document-term matrix is a mathematical matrix that describes the frequency of terms that occur in a collection of documents. In this matrix, each row represents one document and each column represents one term (word).

#### On the equivalence of microbiome and document-term matrix

In the context of microbiome data analysis, we can apply LDA to extract latent bacterial *sub-communities* by mapping a collection of microbiome samples onto a document-term matrix. Accordingly, if we consider that a biological sample corresponds to a document, then the count of a specific OTU in a sample corresponds to the number of occurrences of a specific term in this document. Finally, the common extracted topics are comparable to bacterial sub-communities [27].

Since the mathematical distribution of microbiome data shares several common characteristics with a document-term matrix, such as being high-dimensional, sparse and over-dispersed compositional count data, also, we propose to use LDA to analyze microbiome data. The main advantage of using LDA, in this setting, is that it reduces the dimensions of the dataset from individual OTUs into microbial sub-communities. We defined microbial sub-communities as sets of OTUs that simultaneously occurred across samples. Note that the same OTU can appear in several sub-communities, however the importance of the OTU differs across those sub-communities and the information that each OTU carries might be highly dependent on the other members of a sub-community. If we take an example from a text mining context, we can imagine that the word “rock” does not provide information about the topic of the document itself. However, we can guess the topic of the document using the co-occurring words. On the one hand, “rock” could be associated with “music”, “festival” and “guitar”. On the other hand, it could be associated with “mountain”, “stone” or “mineral”. We suggest that an analogous analysis can be made with microbiome and OTU: a set of co-occurring OTUs is more informative than a single OTU. Finally, all samples share the same set of sub-communities, but they represent those sub-communities in different proportions, where the most important sub-community is the one with the highest proportion, and each sample can be characterized by its important sub-communities.

#### Microbiome theoretical generative process

In this section, we present the LDA generation process for each microbiome in a collection of samples.

For a given number *K* of sub-communities and a given number *V* of OTUs, we propose the following generative model:

$\theta _{s, k} \sim {\mathcal {D}}ir_{K}(\alpha )$, where $\theta _{s, k}$ is the proportion of sub-community *k* in sample *s*.

$\phi _{k,1 \le v \le V} \sim {\mathcal {D}}ir_{V}(\gamma )$, where $\phi _{k,1 \le v \le V}$ is the proportion of OTU *v* in sub-community *k*.

$\alpha \in [0,1]$ the prior weight of sub-communities *k* in a sample.

$\gamma \in [0,1]$ the prior weight of OTU *v* occurring in a sub-communities.

We assume both these parameters are given. The same value of $\alpha$ and $\gamma$ was chosen for each sub-community *k*.

Then, if we denote $w_{i,s}$ the $i^{th}$ read of the $s^{th}$ sample and $z_{i,s}$ its sub-community assignment, they are chosen as follows:

1. $$z_{i,s} \sim {\mathcal {M}}ul(\theta _{s, k}),$$
2. $$w_{i,s} \sim {\mathcal {M}}ul(\phi _{z_{i,s},v}).$$

As mentioned before, the only observable data are OTUs in the collection of samples. Using, LDA we assume that the collection has been generated by the aforementioned steps, where sub-communities and their composition in each sample are latent, i.e. $\phi _{k,v } = \textbf{P}(v | k)$, and $\theta _{s, k} =\textbf{P}(k | s)$ are unknown. To estimate these parameters we used LDA function from topicmodels package (version 0.2-12) R version 4.0.2 (R Development Core Team 2020; R Foundation for Statistical Computing). Furthermore, for the estimation, we chose collapsed Gibbs sampling method in the LDA function.

LDA was run for two different analyses. First, we applied this model to all the microbial niches of one dataset at the same time. The resulting sub-communities were then used as explanatory variables in a predictive model to check if they would recover the original niches. In parallel, we applied the model to each niche separately to describe the microbial composition of a specific niche as well as to assess the relationship between niches.

### Latent Dirichlet allocation in the context of microbiome study

#### Identification of microbial signatures of oral niches

In the same way that LDA can identify documents from the same corpus by identifying common topics, we expect that LDA can recover microbiomes from the same niche by finding similar sub-communities. To illustrate this, we applied LDA on all the niches simultaneously. We fixed the number of sub-communities to K = 5. Then, we evaluated if these obtained sub-communities corresponded to microbial signatures of a specific niche. One way to check this assumption is to assess if we can recover the oral niche the sample originated from by only using the sub-communities resulting from LDA. Finally, we used a predictive model with the sub-communities as explanatory variables and the original niches as response variable. We implemented a multinomial logistic regression model using the nnet R package (see “Datasets” section) with a leave-one-out strategy.

This multi-class model classification was evaluated using several indices such as the F1 score, Cohen’s Kappa score, Matthew correlation coefficient, log loss and multi-class area under the curve (Supplementary Material 1, section 4).

#### The taxonomic structure of each oral niche

In this section, we run the LDA function on each niche individually. The aim here was primarily to reduce the dimensions of a dataset of a microbial niche. To fix the number of sub-communities per niche, we fitted a Dirichlet-Multinomial model between 2 and 8 sub-communities. We chose the number of sub-communities that was minimizing the Laplace approximation of the evidence of the Dirichlet-Multinomial model. We used the dmn function from the DirichletMultinomial package to do so. All niches ended up with a best number of sub-communities between 2 and 4. This was used for both datasets analysed in this work.

The LDA-decomposed dataset was then used to find associations with another oral niche with a method described below.

#### Taxonomic association between different niches

In a study where each individual provides microbiomes from multiple niches, we would like to uncover relationships between microbiomes of different niches. One way of doing that is to study if and how sub-communities of one niche are associated with the OTUs of another niche. With this aim, we propose to test for the association of LDA-derived sub-communities of one niche with all OTUs from another niche using the global test [28]. Note that this test has the ability to test for association between the LDA sub-community and all OTUs in the second microbiome, where the number of OTUs can be larger than the number of samples (*p* $>>$ n). It, therefore, avoids multiple testing correction at the OTU level. The test returns a single *p*-value per pair sub-community - microbiome.

If we denote by $\beta _{j}$ the regression coefficient between the *j*th OTU and the sub-community, to test the association between the OTUs and the sub-community is equivalent to testing the hypothesis:

$$\begin{aligned} H_{0}: \beta _{1} = \beta _{2} = \cdots = \beta _{n} = 0. \end{aligned}$$

The global test proposes to model beta as a random effect, as follows:

$$\begin{aligned} \beta _{1 \le j \le n} \sim {\mathcal {N}}(0, \sigma ^{2}), \end{aligned}$$

and then $H_{0}$ can be re-written as: $H_{0}: \sigma ^{2} = 0$.

Goeman *et al.* [28] show that this hypothesis can be evaluated with the following test statistic:

$$\begin{aligned} Q = \frac{(Y-\mu )^{t}R(Y-\mu )}{\mu _{2}} \longrightarrow {\mathcal {N}}(0, 1), \end{aligned}$$

with *X* the explanatory variables, *Y* the dependent variabe, $R = \frac{1}{n}XX^{t}$, $\mu = g^{-1}(\beta _{0})$ and $\mu _{2} = \textbf{E}(Y^{2} | H_{0})$.

The resulting *p* values were then corrected for multiple testing comparisons across all niches with a Bonferroni correction. As we have a total of *K* sub-communities and a number of niches equal to *m*, all *p* values were multiplied by *Km*. Two oral niches were considered as significantly associated if at least one corresponding *p* value $\le 0.05$ was obtained. Note that the microbiome was transformed with an arcsinus hyperbolic transformation [29]. Finally, while we built our sub-communities with all the available samples, we only kept the samples present in both niches when we ran the association analysis.

**Fig. 1:**
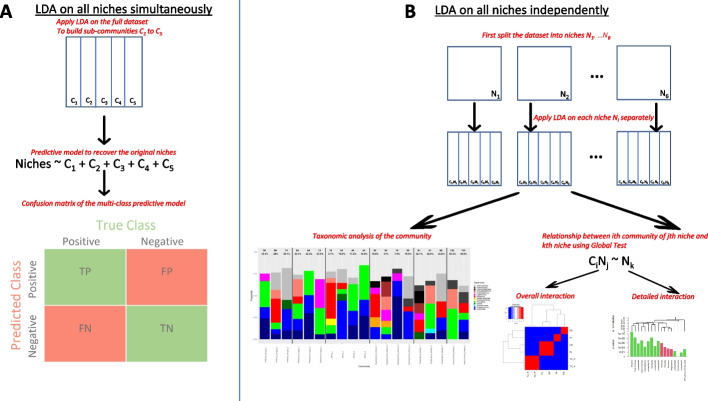
Flowcharts of the methods illustrating the two main analyzes of our method. **A** LDA applied on all the niches simultaneously to find microbial signatures. **B** LDA applied on each niche separately to find taxonomic structures and assess relationships between niches

The complete flowchart of our method can be found in Fig. 1.

### Choosing the right number of sub-communities *K*

When using LDA, the number of sub-communities needs to be chosen. While several methods exist to do this [30–34], there is no consensus about which one is the best. We have used different methods, as described below.

For the analysis per niche (for both doda and HMP), we applied the method used in DMM for fixing the number of sub-communities and then by checking the composition of the sub-communities and (potentially) increasing or decreasing this number if some sub-communities seemed questionable. This method minimizes the Laplace approximation of the evidence of the DMM model with respect to the number of sub-communities *K*.

When analysing all niches simultaneously, the method used by DMM was used to yield an upper bound in the number of sub-communities. More details are given in the Results section.

### Datasets

#### Dutch Oral Dataset (doda)

In a previous project [35], microbial samples were collected from 268 healthy, young (18–32 y.o.) Dutch adults after overnight fasting and restraining from any oral hygiene for 24 h. The details about the inclusion and the exclusion criteria are described in [36]. DNA was extracted from the samples and processed for amplicon sequencing by barcoded sequencing of the V4 hypervariable region of the 16S rRNA gene on the Illumina MiSeq platform.

The complete dataset was then randomly subsampled to 10,000 reads per sample, and the subsampled data is available as Additional file 3. A total of 1474 samples from 6 different oral niches (unstimulated saliva (US), anterior tongue (TCA), posterior tongue (TCP), supragingival plaque (PL), subgingival plaque (PS) and interproximal plaque (PI)) and 1188 OTUs (Operational Taxonomic Units) remained. The abbreviations (US, TCA, TCP, PL, PS and PI) will be used later in the paper. The unstimulated saliva microbiome dataset has already been analyzed [35]. Although saliva is not a niche as such, we will use this term in this paper for convenience.

#### HMP dataset

A publicly available dataset from the Human Microbiome Project (HMP) [37, 38] was used to validate our findings. This dataset was already analyzed to assess the relationship between niches and OTUs [21, 39]. We then proposed to analyze this dataset as well to compare our results. The Human Microbiome Project carried out three phases of sequencing the 16S rRNA gene, which was performed using the 454 Titanium sequencing platform. For the datasets used here, the V3–V5 hypervariable region of the 16S rRNA gene was sequenced.

This dataset was first subsampled to 3000 reads. The remaining dataset consisted of a total of 5637 microbial samples and 15936 OTUs coming from 18 different niches (anterior nares, buccal mucosa, hard palate, keratinized gingiva, left antecubital fossa, left retroauricular crease, mid vagina, palatine tonsils, posterior fornix, right antecubital fossa, right retroauricular crease, saliva, stool, subgingival plaque, supragingival plaque, throat, tongue dorsum and vaginal introitus).

Some oral samples had a large proportion ($\ge 80\%$) of OTU 001 (*Propionibacterium*). While this genus is common in the skin microbiome, it is usually not present in the oral microbiome in a large proportion (more than 1%) [40]. As its presence at more than 1% is therefore likely to be due to contamination, we excluded individuals with more than 2% of *Propionibacterium* in one of their oral niches from the entire analysis. The list of the excluded sample IDs can be found in Additional file 1, section 2.

This dataset was obtained from samples collected at 3 different visits. We only analyzed the samples of the first visit. We had a total of 2501 samples for this first visit after removing the sample IDs as indicated above.

### Software

All analysis and figures were produced using R 4.0.2 (on a PC with Windows 10 operating system) and using the following R packages: globaltest 5.44.0, survival 3.3-1, dplyr 1.0.9, pROC 1.18.0, MLmetrics 1.1.1, caret 6.0-93, lattice 0.20-45, forcats 0.5.2, stringr 1.4.1, tidyr 1.2.0, nnet 7.3-17, tidytext 0.3.3, gplots 3.1.3, microbiome 1.12.0, ggplot2 3.3.6, phyloseq 1.34.0, DirichletMultinomial 1.32.0, IRanges 2.24.1, S4Vectors 0.28.1, BiocGenerics 0.36.1, topicmodels 0.2-12. All random seeds used to run LDA were fixed to 2022.

## Results

### LDA is able to identify discriminatory signatures of microbial niches

We first ran LDA on all niches from the Dutch Oral Dataset (doda) dataset simultaneously. The aim here was to see if the resulting sub-communities can help assess (dis)similarities between oral niches. We run this analysis with a fixed number of 5 sub-communities.

**Fig. 2:**
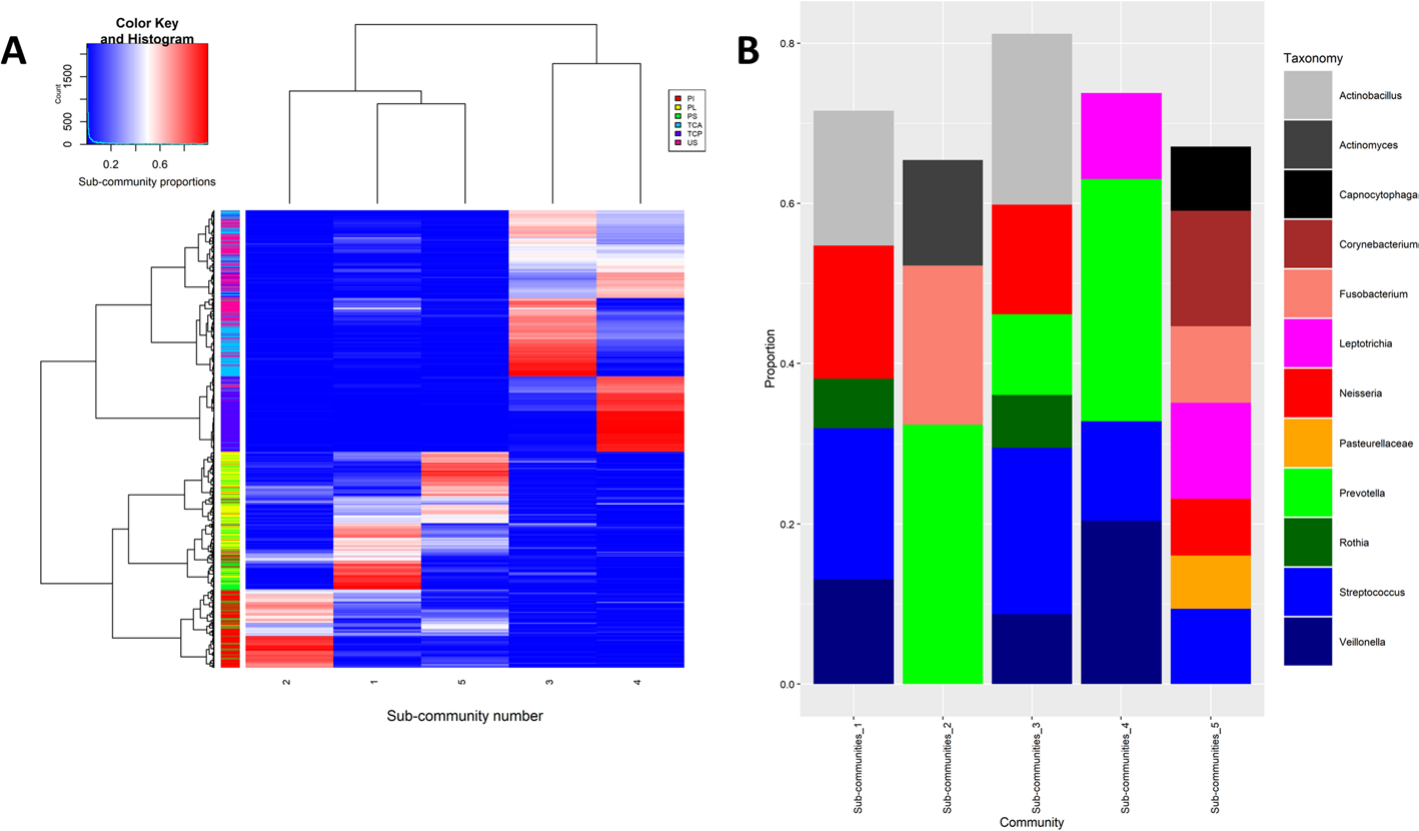
Distribution and composition of the microbial signatures. **A** Heatmap of the proportion of the sub-communities from LDA across samples of the doda dataset. Sub-communities are the column, samples are the rows. The proportion of each sub-community is clearly discriminated by the original oral niche. **B** Microbial signature composition. Only genera (or higher level taxa) that represent at least 5% of the sub-community are plotted here. PI, Interproximal Plaque; PL, Supragingival Plaque; PS, Subgingival Plaque; TCP, Tongue Coating Posterior; TCA, Tongue Coating Anterior; US, Unstimulated Saliva

The sub-communities were able to discriminate the original microbial oral niches (Fig. 2). For example, the posterior tongue microbiome (TCP) is almost only composed of sub-community 4, while the interproximal plaque (PI) is mainly formed of sub-community 2. In addition, those sub-communities almost did not appear in the composition of any other oral niche, suggesting that they represent unique microbial signatures for their corresponding niches. Still from Fig. 2, we can see two main groups of microbial oral niches sharing more similarities. On the one hand, the microbiomes that come from different plaque niches (supragingival—PL, subgingival—PS, and interproximal—PI) are mostly composed of sub-communities number 1, 2, and 5. On the other hand, the salivary and tongue (anterior—TCA and posterior—TCP) microbiomes are mainly composed of sub-communities 3 and 4. Interestingly, using principal component analysis, the same separation of plaque and “mucosal” samples is obtained (see Additional file 1, Section 3, Figures S7 and S8), although using PCA the differences between niches within one group are much less clear using the first two PCs. Specifically, the fact that the posterior tongue microbiome (TCP) signature is distinct from those of other niches is not apparent. The same is true of the interproximal plaque (PI) microbiome. Detailed heatmaps per niche can be found in Additional file 1 (Section 1, Figures S1 to S6).

**Fig. 3:**
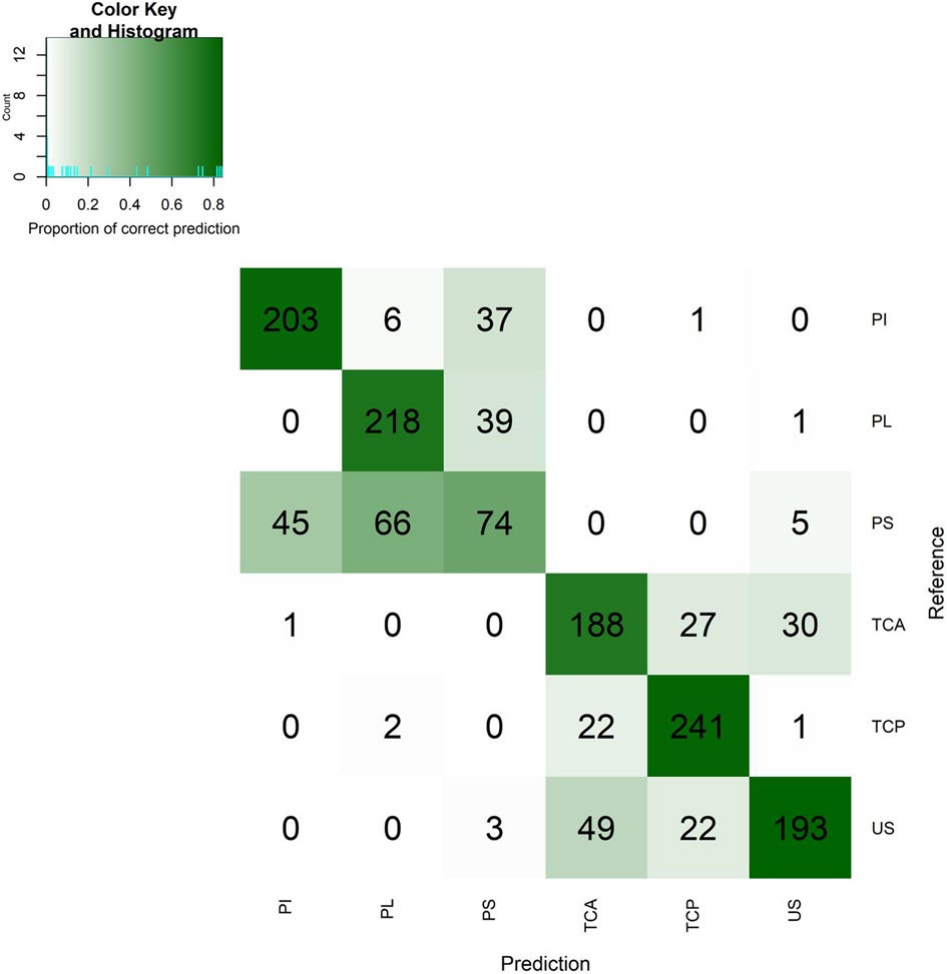
Confusion matrix of the predictive model applied to the doda dataset. Rows correspond to the reference, and columns to the predicted values. For example, among the 241 anterior tongue samples, 190 were accurately predicted, 25 were wrongly predicted as posterior tongue samples and 30 were wrongly predicted as unstimulated saliva. Niche labels: see abbreviations

We have also quantified how discriminative sub-communities are of their corresponding niches, for all niches, by fitting a multinomial logistic regression model to the niche (response) using the sub-communities as predictors (explanatory variables). This was done using a leave-one-out cross-validation strategy to recover the original oral niche of a sample, using data from all remaining samples. The global accuracy was 76%, and per niche sensitivities ranged between 39 and 90%, with the latter obtained for the posterior tongue (TCP; Fig. 3). Almost all oral niches were predicted with accuracy above 76%, except for the subgingival plaque (PS—39% accuracy, also see “Heuristic interpretation” section).

### Latent Dirichlet allocation can describe the taxonomic structure of microbial niches

We also ran LDA on each oral niche individually with two main aims. Firstly, we wanted to examine the taxonomy of each sub-community and possibly identify patterns. Secondly, we wanted to assess if sub-communities contain all information from OTUs pertaining association between niches. We will look in this subsection at the first point, whilst the second will be handled in the next subsection.

**Fig. 4:**
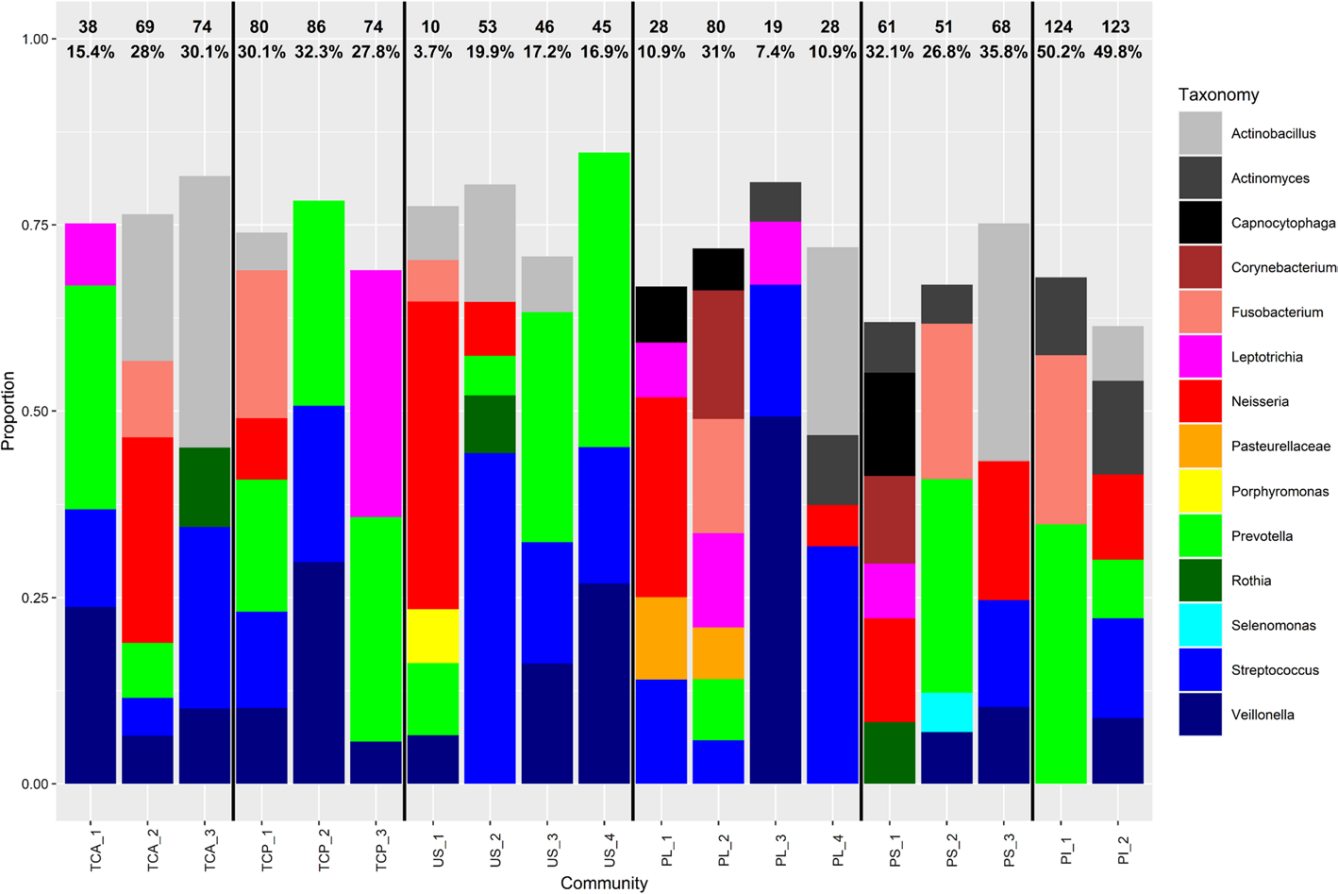
Taxonomic proportion for each sub-community in the doda dataset. While the sub-communities have been built on OTU level, we plot here the lowest taxonomic level available for each OTU. Only the taxa that contributed to at least 5% of a sub-community are present here. The numbers and the percentage at the top of the stacked bar plot respectively correspond to the number and the proportion of samples composed of at least 50% of this sub-community. Niche labels: see abbreviations

From the taxonomic composition of the sub-communities (Fig. 4), we saw that the “mucosal” samples (saliva and tongue samples—US, TCP, TCA) globally shared more similarities regarding their taxonomy. In particular, the combination *Prevotella-Streptococcus-Veillonella* was present in several sub-communities. Moreover, these niches seemed to have lower diversity within their sub-communities. The low abundant taxa were indeed less present in mucosa-derived than in the plaque samples (PI, PL and PS).

In contrast, the plaque niches (PI, PL and PS) shared a greater diversity and an over-representation of the *Corynebacterium-Capnocytophaga* as well as *Actinomyces-Fusobacterium-Prevotella* combinations.

We conclude that LDA can highlight similarities between niches regarding their taxonomic compositions.

### Association between LDA sub-communities and microbiomes

Here, we assess if sub-communities contain all information from OTUs pertaining association between niches. To check this, we test for association between two niche-specific microbiomes using sub-communities of one niche and all OTUs of the other niche. Firstly, we use LDA to decompose each niche-specific microbiome into sub-communities. Secondly, we test the association between each sub-community obtained with another niche’s microbiome by using the global test [28]. For example, to test if there is an association between the unstimulated saliva (US) and supragingival plaque (PL) microbiomes, we first extracted sub-communities and their proportions using saliva samples only. We then applied the global test using each extracted sub-community as response, and the supragingival plaque microbiome OTUs as explanatory variables. We repeated this step using each of the four salivary sub-communities as response in turn, and applied a Bonferroni correction on the obtained global test *p* values. Saliva and supragingival plaque microbiomes were considered to be significantly associated if at least one of the obtained global test *p* values was below 0.05. We performed this analysis for all pairs of niches, and considered the microbiomes of two niches to be associated if at least one global test remained significant (after Bonferroni correction).

**Fig. 5:**
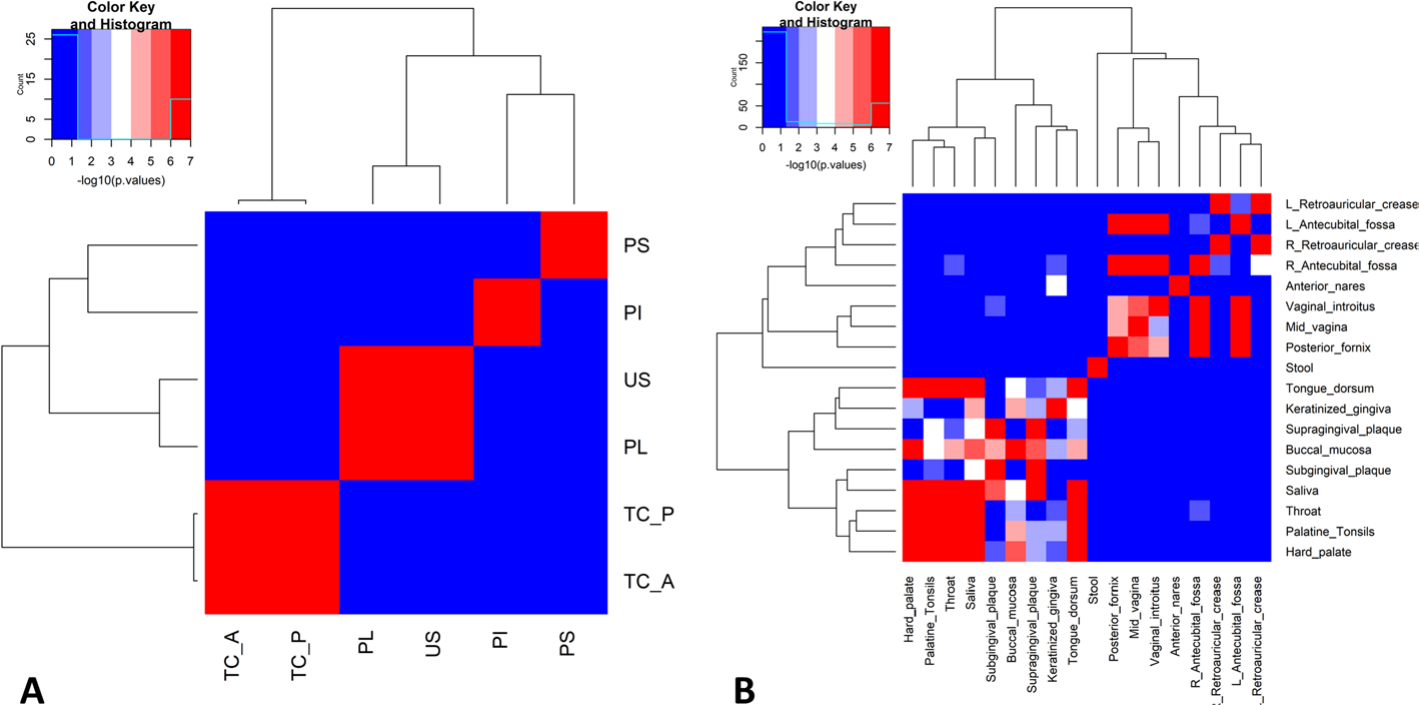
Heatmap of the negative logarithm of the *p* values of the relationship between the niches. For all the sub-communities versus microbiome tests the lowest *p* value is used. doda (left) and hmp (right) datasets. Niche labels: PS Subgingival plaque; PI Interproximal plaque; US Unstimulated saliva; PL Supragingival plaque; TCP Posterior tongue; and TCA Anterior tongue

First we noticed that, if one sub-community of a niche displayed association with all OTUs of a second niche, all other sub-communities also displayed association, according to the global test (Additional file 2). This is a result of the compositional aspect of sub-communities. For example, our approach identified associations between posterior and anterior tongue microbiomes, as well as between unstimulated saliva and supragingival plaque microbiomes (Fig. 5). The *p* values reported in Fig. 5 are the lowest for each pairwise comparison.

Secondly, both heatmaps in Fig. 5 display a striking symmetry. For example, we found that saliva sub-communities were associated with the supragingival microbiome OTUs, as well as supragingival sub-communities were associated with saliva OTUs. This symmetry confirms the ability of LDA summarizing the information contained in OTUs: testing for association between sub-communities of niche 1 on OTUs of niche 2 leads to the same conclusions as testing for association between sub-communities of niche 2 on OTUs of niche 1.

The complete table of the *p* values for doda can be found in Additional file 2.

We validated these findings using the Human Microbiome Project (HMP) dataset, consisting of 18 different niches of the body (see “HMP dataset” section for more details). Using LDA with 3 sub-communities per niche (see “Choosing the right number of sub-communities *K*” section), we found that the niches sharing more biological similarities were usually significantly associated. For example, we can see in the right panel of Fig. 5 that almost all oral niches are significantly associated with each other. The same pattern can be observed for the vaginal niches.

Both of these results illustrate that, and how, niche-specific sub-communities report relevant information, enabling them to replace the OTUs in analyses in a consistent way.

### Heuristic interpretation

In the subsection “LDA is able to identify discriminatory signatures of microbial niches”, we extracted microbial signatures for each oral niche. Further examination showed that the niches were almost never composed by a single sub-community, but rather by a mix of several ones. We observed that a single niche tends to be represented by a main sub-community and one or several minor sub-communities. The unstimulated saliva (US) microbiome provides a good example (Fig. 2). While sub-communities 3 and 4 are clearly dominant in the salivary microbiome, sub-community 1 (much more abundant in the plaque samples) is still present at a non-negligible proportion in many samples (9.3% on average). This finding is in line with the general knowledge that the unstimulated salivary microbiome is a mix of the niches in the oral ecosystem [41], here a mix of tongue and plaque microbiomes.

Let us now look at the results for subgingival plaque, the only niche with a high proportion (Fig. 2) of 3 different sub-communities (1, 2, and 5), which was often misclassified by our prediction model. This may partly be explained by the fact that these sub-communities were largely present in the other plaque samples (supragingival and interproximal). One possible explanation for the lower predictive accuracy for this subgingival niche is that it is difficult to sample subgingival plaque from healthy individuals, as was the case here since there would not be attachment loss or periodontal pockets. Thus, subgingival plaque sample was most likely polluted with supragingival or interproximal plaque.

**Fig. 6:**
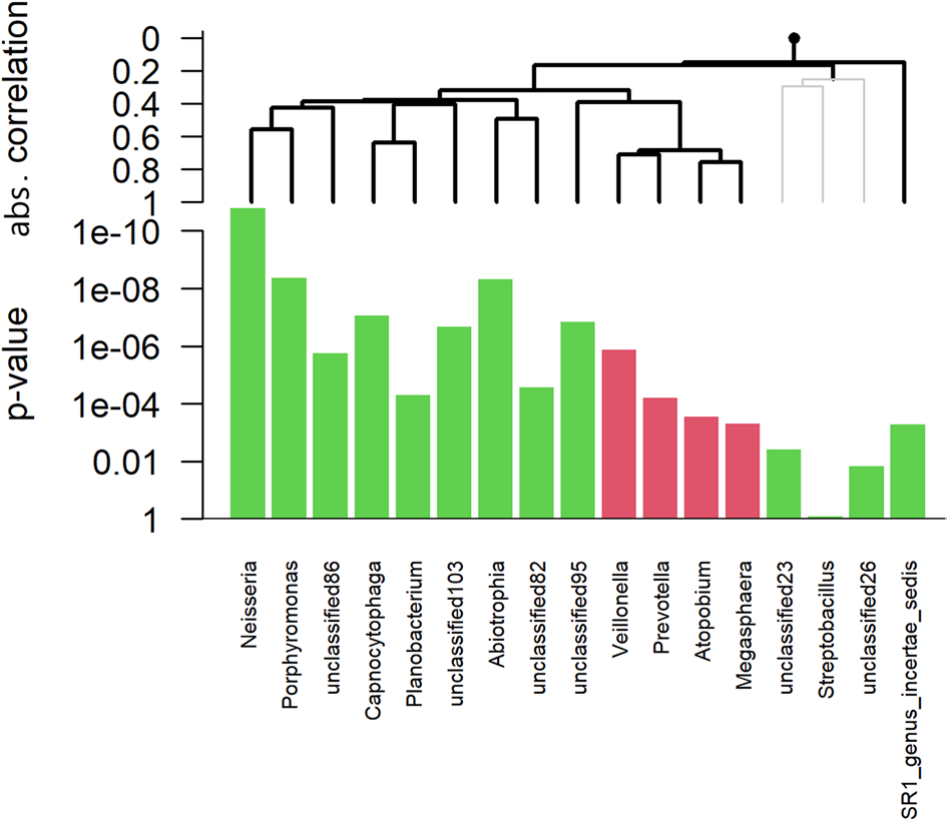
Output of the global test between the first supragingival plaque sub-community and salivary microbiome. Green corresponds to a positive association and red to a negative one. Here, the presence of the genus *Neisseria* in the saliva is positively significantly (*p* value $\le 1e^{-10}$) associated with the development of the first supragingival plaque sub-community. We performed a Genus-level summary before to plot this output to have a clearer view of the sub-communities-genus dependency

We can also examine associations in more detail. For example, we have found the supragingival plaque microbiome composition to be associated with that of the unstimulated saliva microbiome (Fig. 5). A global test output between the supragingival plaque sub-community 1 and the salivary microbiome shows that this sub-community is positively associated with genera such as *Neisseria* and *Capnocytophaga*, greatly abundant in this supragingival plaque sub-community (Fig. 6). On the other hand, genera that were negatively correlated with this sub-community were absent in this supragingival plaque sub-community.

Finally, we note that our results are consistent with those obtained by others. The salivary microbiome used here was previously analyzed in [35]. There, five different ecological states were proposed depending on the microbiome composition, based on a spectral clustering method [Figure 1 in [35]]. In spite of our methods being mathematically very different, we obtained a significant association between the clusters derived from [35] and the proportions of our 5 sub-communities (Kruskal-Wallis test between the proportion of salivary sub-communities in samples and the clusters from, *p* value $\le 10^{-16}$).

### Choosing the right number of sub-communities (cont.)

When we analyzed the 6 niches simultaneously of the doda dataset, the best number of sub-communities according to the DMM model was 12. These 12 LDA-yielded sub-communities were however not easy to interpret. For example, several sub-communities had a unique genus representing more than 80% of the total composition (Additional file 1, section 5, Figure S9). In addition, some sub-communities appeared to be evenly spread across all niches, rather than being concentrated in one or two specific niches. Finally, by reducing the number of sub-communities we realized that these unspecific sub-communities merged into other sub-communities, usually representative of a specific niche. As we were aiming to extract bacterial signatures discriminating different oral niches, we decided to choose a more parsimonious model with a smaller number of sub-communities, closer to the observed number of niches. Hence, for our analyses we extracted $K=5$ sub-communities and used them in our predictive model. This yielded sub-communities that were associated with biologically related (OTU-based) microbiomes, as we saw in Fig. 5, in both datasets considered.

It appeared to us that $K = 5$ provided the most meaningful sub-communities. This is for example the highest number of sub-communities so that there is no overlapping on the two-dimensions visualization of the LDAvis package. Moreover, this number of sub-communities is close to our number of niches which helped us to assess the sub-communities - niches relationships.

To give us an idea about the impact of the number of communities chosen, we compared original niche predictions obtained with $K=5$ with those using $K=12$. The global accuracy of the prediction model was naturally higher when $K=12$ was used: 87%, compared with 76% with $K=5$ used. However, this increase in predictive power comes with a loss of interpretability individual sub-communities often represent a single sample, making it harder to understand relationships between microbiomes.

Finally, we run a simulation study to check how the method used by DMM to estimate the number of sub-communities changes with the number of samples available. For this, we created 20 simulated datasets using the LDA generative process with a true number of sub-communities $K = 5$ and a total number of samples $n = 1474$. We then applied the dmn function on each dataset and varied the number of included samples between $n = 100$ and $n = 1474$ for each case. We found that the number of estimated sub-communities by the dmn function is highly dependent on the number of included samples. This number varied from $K_{est} = 15$ with $n = 1474$ to $K_{est} = 5$ with $n = 5$. This information encourages us to use a lower number of sub-communities than the one proposed by dmn when the number of samples is high. The detailed results of this simulation study can be found in Additional file 1, section 6, Figures S10 to S12.

## Discussion and conclusion

In this study, we proposed to use Latent Dirichlet Allocation to reduce dimensions of multi-niche microbiome data. LDA is an unsupervised machine learning technique able to jointly analyze microbiomes coming from several niches. This method allows us to bring out microbial signatures of those niches, taking into account the multinomial and compositional nature of the data.

Using our approach on multiple microbial niches simultaneously, we have been able to better describe associations between them, by identifying niche-specific microbial signatures. These signatures can also be used to predict the original niche of a sample. We also illustrated that when analysing data from individual niches, sub-communities revealed the latent taxonomic structure of microbiomes and were used to assess the relationship between several niches.

In particular, when analysing multiple microbial niches in the oral cavity from the Dutch Oral Dataset, we have found that the salivary microbiome is related to supragingival plaque, but also that the salivary microbiome is sharing microbial signatures with the tongue and (in a lower proportion) supragingival plaque. However, tongue microbial environments show relatively little association with those of plaque niches. The unstimulated saliva, often used to represent the entire oral cavity, has its sub-communities mostly related to that of supragingival plaque, and displays in fact little relation to other oral microbial niches. This result can, however, no longer be observed in the HMP dataset. In this dataset, saliva samples were collected by a mixture of stimulated and unstimulated procedures which result in different microbial compositions [42]. Stimulated saliva usually has a higher microbial diversity which might lead to more association with other oral niches.

Our method requires a number *K* of sub-communities to be given. In applications, where possible, we used *K* estimated by the DMM method [20]. However, we found evidence that this estimate varies with the number of samples available, in a way that the resulting ${\hat{K}}$ is not as small as it could be, making interpretation more difficult. A better method to choose optimal *K* would be useful in this context. This problem is however complex, in this context where a mixture between a Dirichlet and a multinomial is involved.

Dimension reduction techniques, such as Principal Component Analysis (PCA), are widely used to analyze microbiome data. PCA has been used to visually discriminate microbiomes from several niches as well as overtime [18]. In that work, PCA was able to discriminate microbial niches, but the principal components extracted were difficult to interpret biologically. Moreover, PCA is built to handle continuous data, while microbiome data are composed of counts and involve compositionality, sparsity and overdispersion, all of which are not well handled by PCA. PCA, therefore, would require at least a data transformation before it can be applied to microbiome data. LDA, on the other hand, is suited to the inherent properties of microbiome data and can also capture non-linear relationships.

Having said that, it has been shown that LDA can be interpreted as a multinomial PCA model, and therefore it can be seen as a discrete analogue to PCA [43].

Dimension reduction can also be achieved using a Dirichlet Multinomial Mixture (DMM), which has already been used with the same aim (decompose microbiome into sub-communities and assess the relationship between microbial niches) [19–21]. LDA and DMM are mathematically similar and yet present important differences. The main difference is related to the nature of sub-communities. Indeed, the DMM model assumes that every microbial sample belongs to a single sub-community. In contrast, using LDA each sample is by definition a mixture of several sub-communities with a different composition so that, even if the samples share some sub-communities, the proportion of the sub-communities differs across them. This aspect of LDA preserves the uniqueness of each sample. In the context of text-mining, DMM is often used to analyze short text such as tweets or GoogleNews [44], and in such cases, a single topic per document seems likely. In our case, however, since microbiomes are highly complex with several higher and lower abundant species, this assumption does not seem plausible enough to be considered. Moreover, we wanted to check the reliability of our sub-communities by using the sub-community proportion per sample as an independent variable in our predictive model. If each sample belongs to a single sub-community though, prediction can no longer be done using cross-validation.

An LDA sub-community can be defined as a repeating pattern of co-occurring OTUs in a collection of samples (niches/collection of niches). Note that, as an unsupervised method which does not use sample labels, it is up to the user to gain insight from sub-communities. We have here illustrated that, by examining the association of sub-communities and other microbiomes, further insight into their interpretation can be gained.

Here we point out that the nature of the resulting sub-communities is dependent on both the data collection process and the number of sub-communities used. Like any other data analysis approach, pre-processing is a crucial step. For example, when performing topic modeling in the context of text mining, pre-processing involves eliminating words that are commonly used and therefore carry little useful information, for example, common verbs, articles, or punctuation. Further research on the impact of this pre-processing on the microbiome needs to be performed in the future.

To conclude, to analyse multiple microbial niches we proposed to use Latent Dirichlet Allocation. We have shown that bacterial signatures yielded can be used to discriminate as well as characterize niches, and to predict the original niche of a microbial sample. Sub-communities can replace microbial OTUs in data analyses, yielding similar conclusions which are potentially easier to interpret due to dimension reduction. Finally, by taking into account multiple microbial niches, the analysis can shed light into relationships between niches, and better inform the choice of a single niche to represent them all, if need be.

### Supplementary information

**Additional file 1**. Supplementary figures, details of excluded samples (HMP dataset), evaluation of the predictive model and simulation study details.

**Additional file 2.** P-values table for section 3.3.

**Additional file 3.** The subsampled DODA dataset.

## Data Availability

The Dutch Oral dataset we used to run this study is provided as Additional file 3.

## Abbreviations

OTU: Operational taxonomic unit
ANOSIM: Analysis of similarity
PCA: Principal component analysis
DMM: Dirichlet multinomial model
LDA: Latent Dirichlet allocation
HMP: Human Microbiome Project
DODA: Dutch Oral DAtaset
PI: Interproximal plaque
PL: Supragingival plaque
PS: Subgingival plaque
TCP: Tongue coating posterior
TCA: Tongue coating anterior
US: Unstimulated saliva
