# Supplementary material for: A new approach to describe the taxonomic structure of microbiome and its application to assess the relationship between microbial niches
Source: BMC Bioinformatics. 2024 Feb 5;25:58. doi: 10.1186/s12859-023-05575-8 (PMC10840258; doi:10.1186/s12859-023-05575-8)
Supplement: Supplementary file 1 — Additional file 1. Supplementary figures, details of excluded samples (HMP dataset), evaluation of the predictive model and simulation study details. [file 12859_2023_5575_MOESM1_ESM.pdf]

# Supplementary materials: A new approach to describe the taxonomic structure of microbiome and its application to assess the relationship between microbial niches

Vincent Y. Pappalardo[1,2], Leyla Azarang[2], Egija Zaura[1], Bernd W. Brandt[1], and Renee X. de Menezes[2]

[1]Department of Preventive Dentistry, Academic Centre for Dentistry Amsterdam, University of Amsterdam and Vrije Universiteit Amsterdam, The Netherlands
[2]Antoni van Leeuwenhoek Hospital, Biostatistics Centre, Department of Psychosocial Research and Epidemiology, Netherlands Cancer Institute, Amsterdam, The Netherlands

September 28, 2023

## 1 Heatmap Details

This section presents a zoom-in of figure 2 of the paper. This provides us a better view of the distribution of sub-communities within one niche.

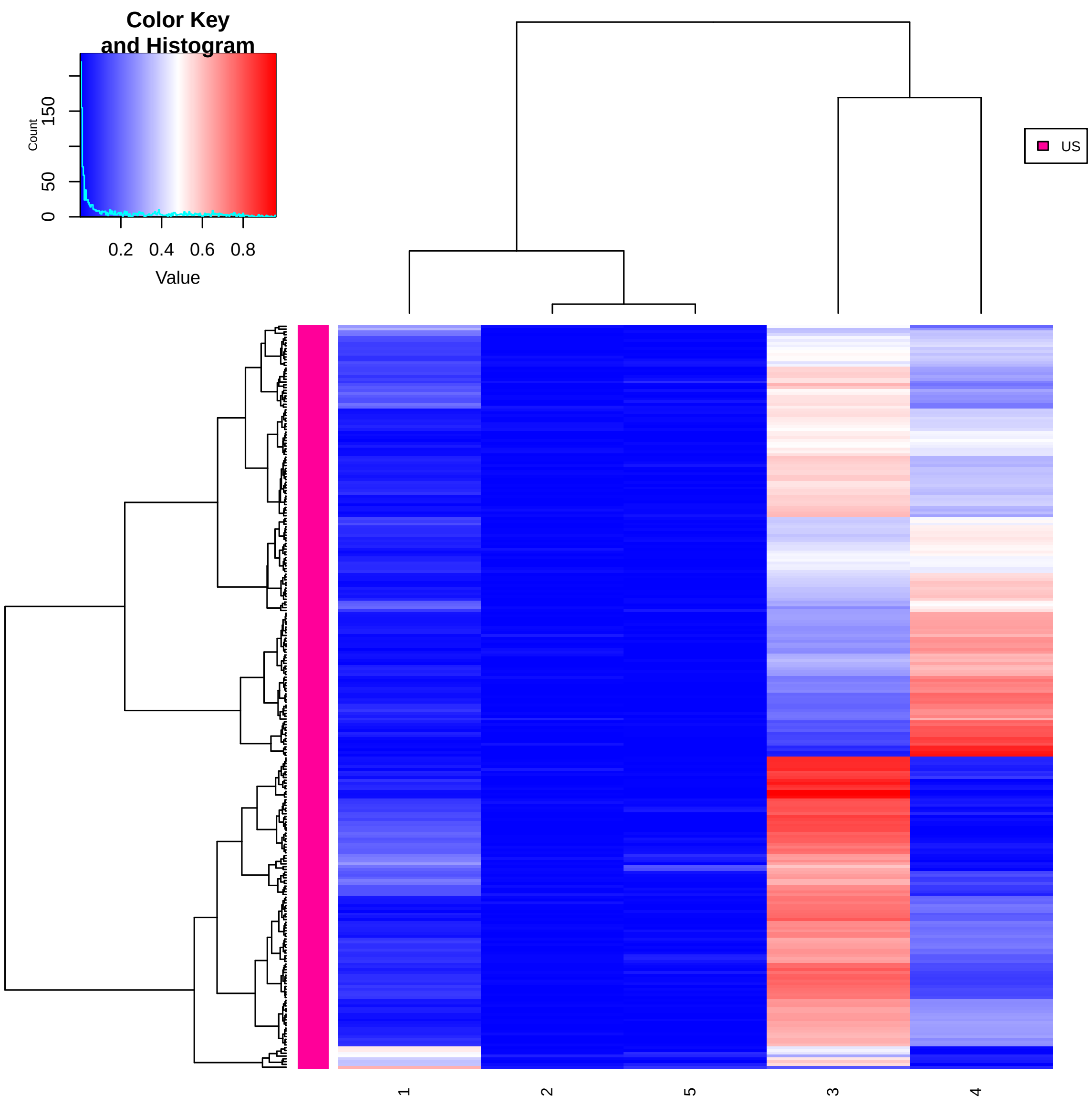


Figure S 1: **Details of the distribution of the sub-communities within the unstimulated saliva.** This niche is mainly composed of sub-communities 3 and 4. Sub-community 1 is also present in a smaller proportion.

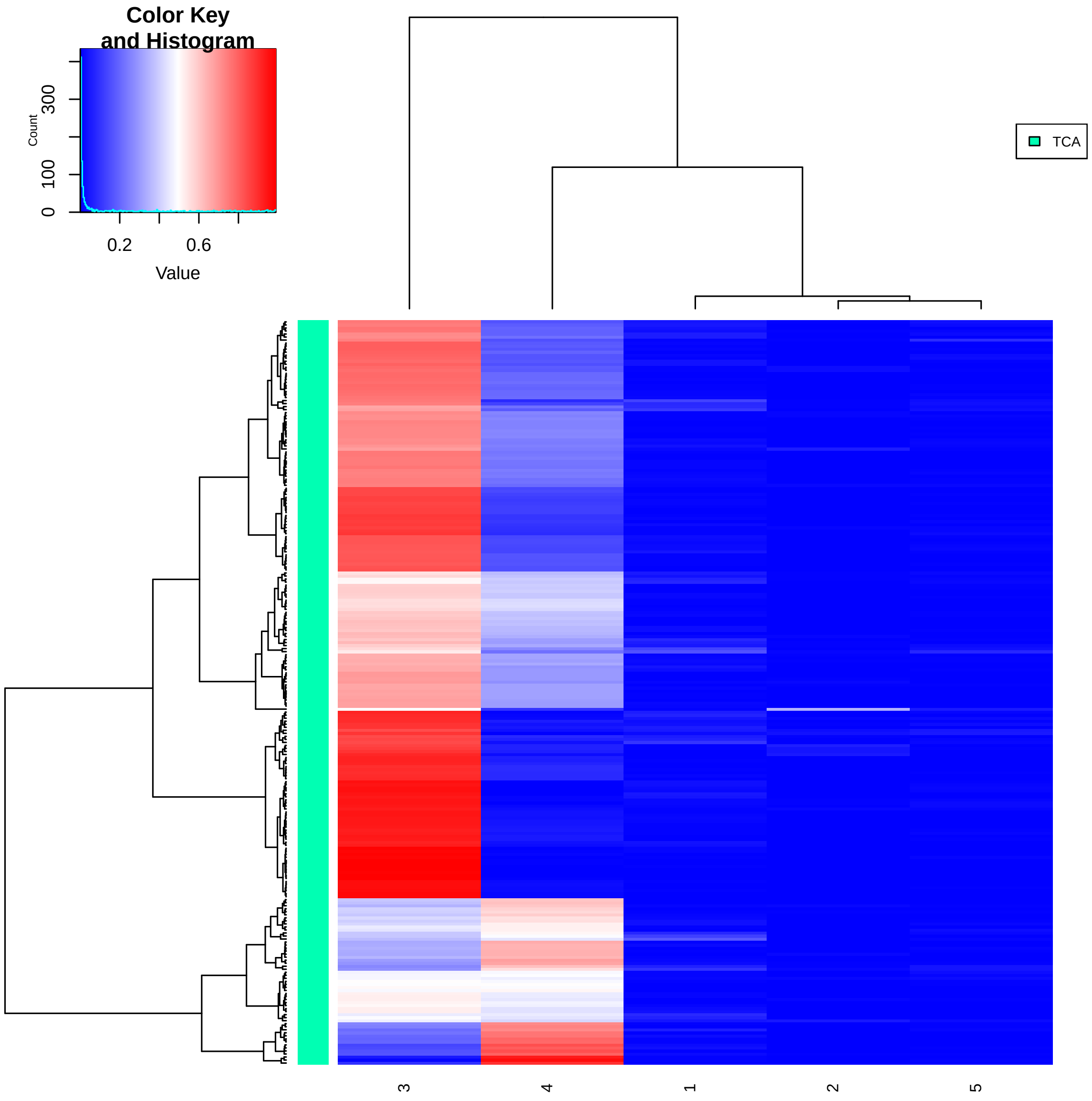


Figure S 2: **Details of the distribution of the sub-communities within the anterior tongue.** This niche is mainly composed of sub-community 3. Sub-community 4 is also present in a smaller proportion.

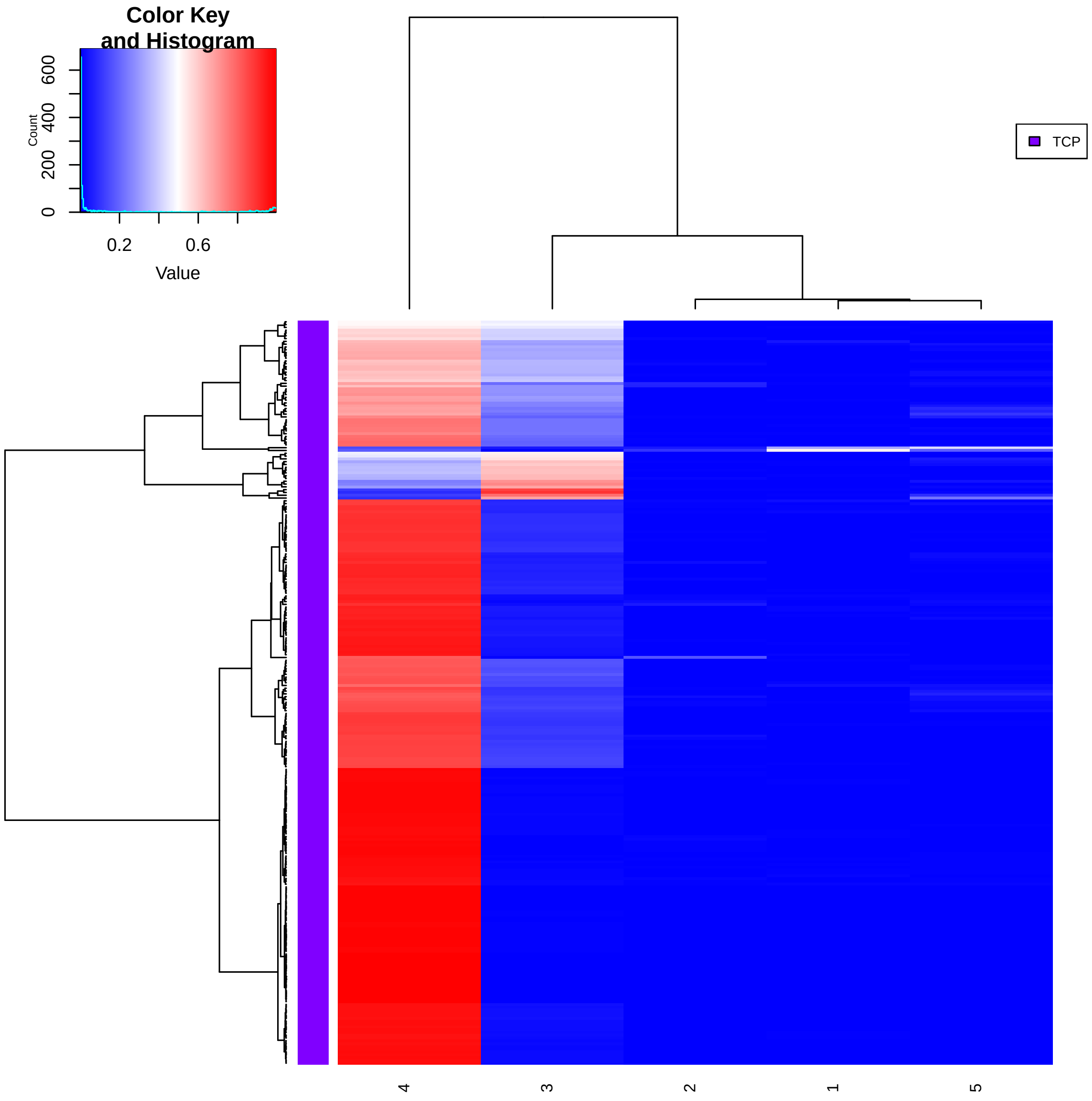


Figure S 3: **Details of the distribution of the sub-communities within the posterior tongue**. This niche is mainly composed of sub-community 4. Sub-community 3 is also present in a smaller proportion.

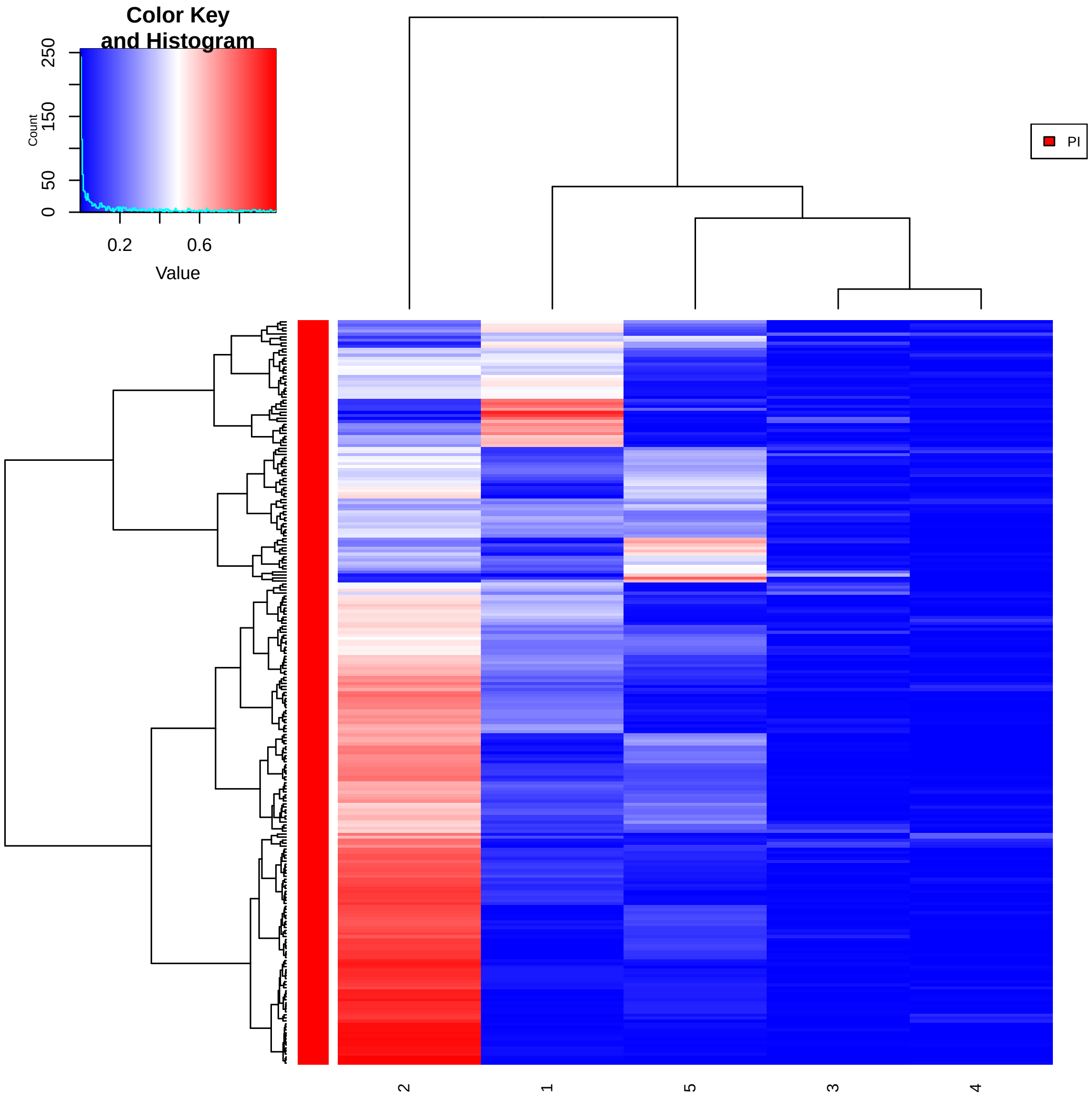


Figure S 4: **Details of the distribution of the sub-communities within the interproximal plaque.** This niche is mainly composed of sub-community 2. Sub-communities 1 and 5 are also present in a smaller proportion.

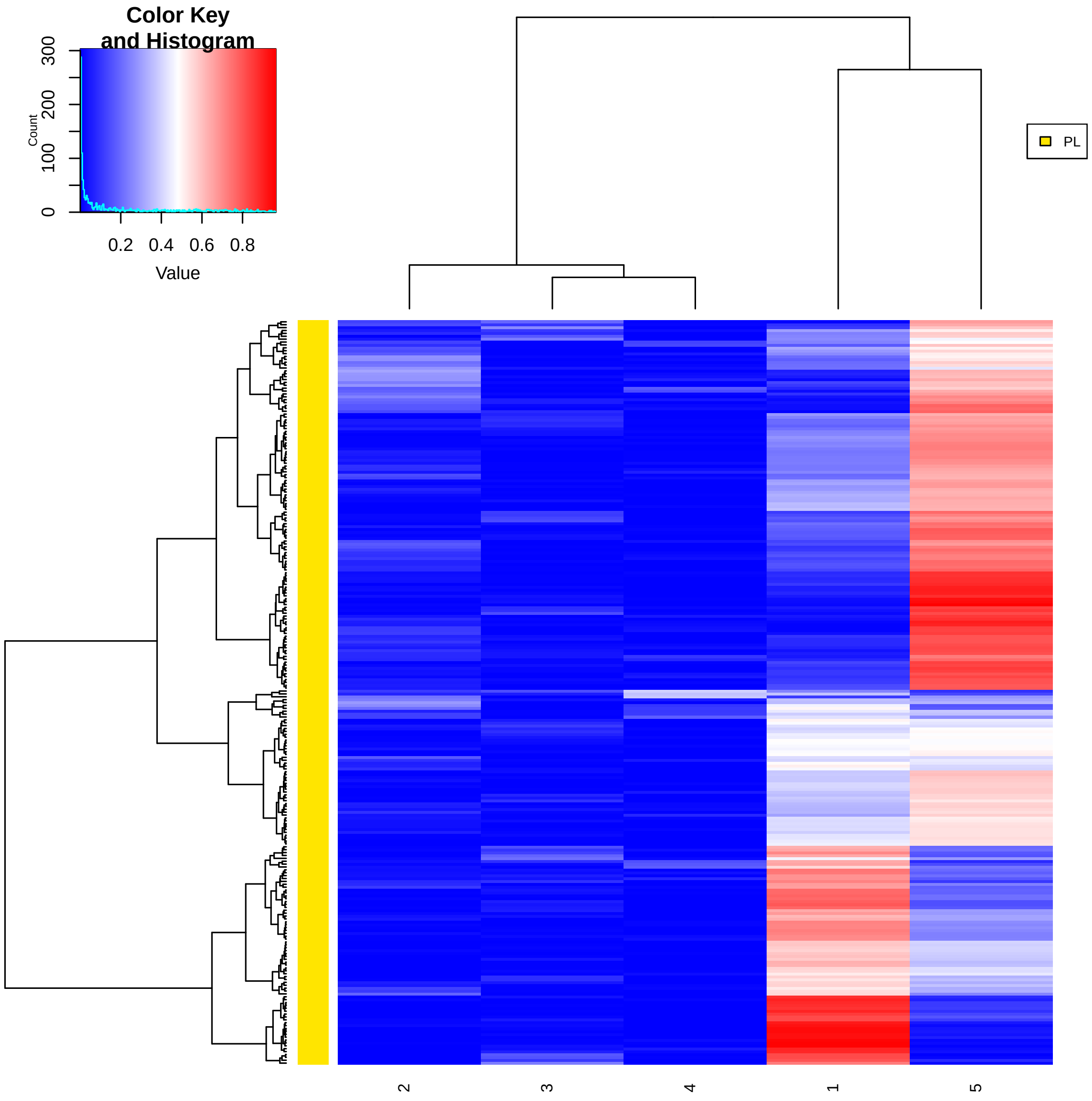


Figure S 5: **Details of the distribution of the sub-communities within the supragingival plaque.** This niche is mainly composed of sub-community 5. Sub-community 1 is also present in a smaller proportion.

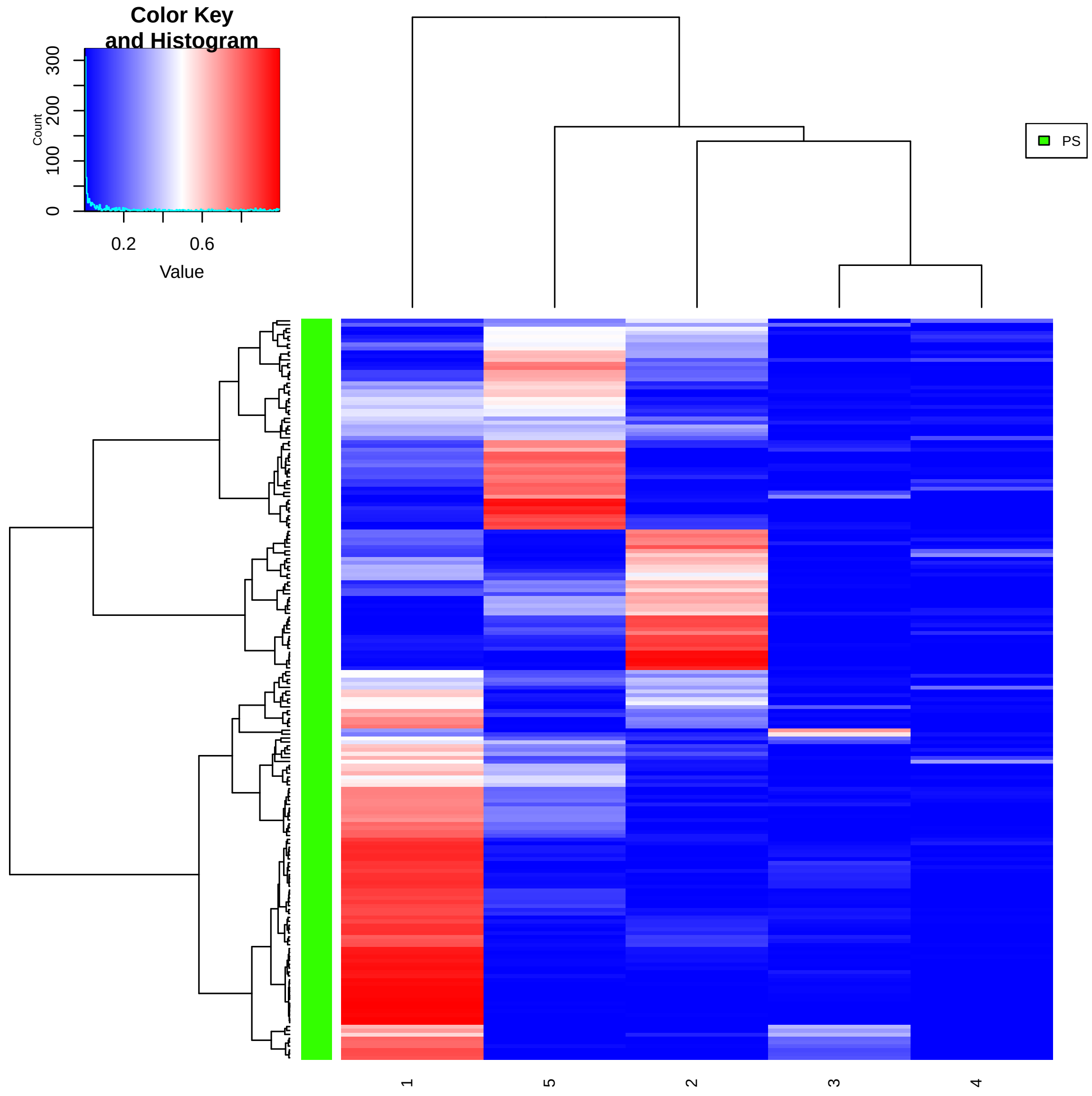


Figure S 6: **Details of the distribution of the sub-communities within the subgingival plaque.** This niche is mainly composed of sub-community 1, 2 and 5.

## 2 Excluded sample ID HMP dataset

This section present the list of the excluded ID in the Human Microbiome Project dataset. Those samples present a dubious oral microbiome composition since they had a big proportion of OTU001 *Propionibacterium*, a bacteria very common in the skin microbiome but usually very rare in the oral microbiome. All the samples coming from the following IDs were therefore excluded from the enire analysis.

List of excluded IDs: 103092734, 250593475, 561079058, 663835652, 682102541, 739574095, 765034022, 875002022, 901775393, 938202701.

# 3 PCA plots

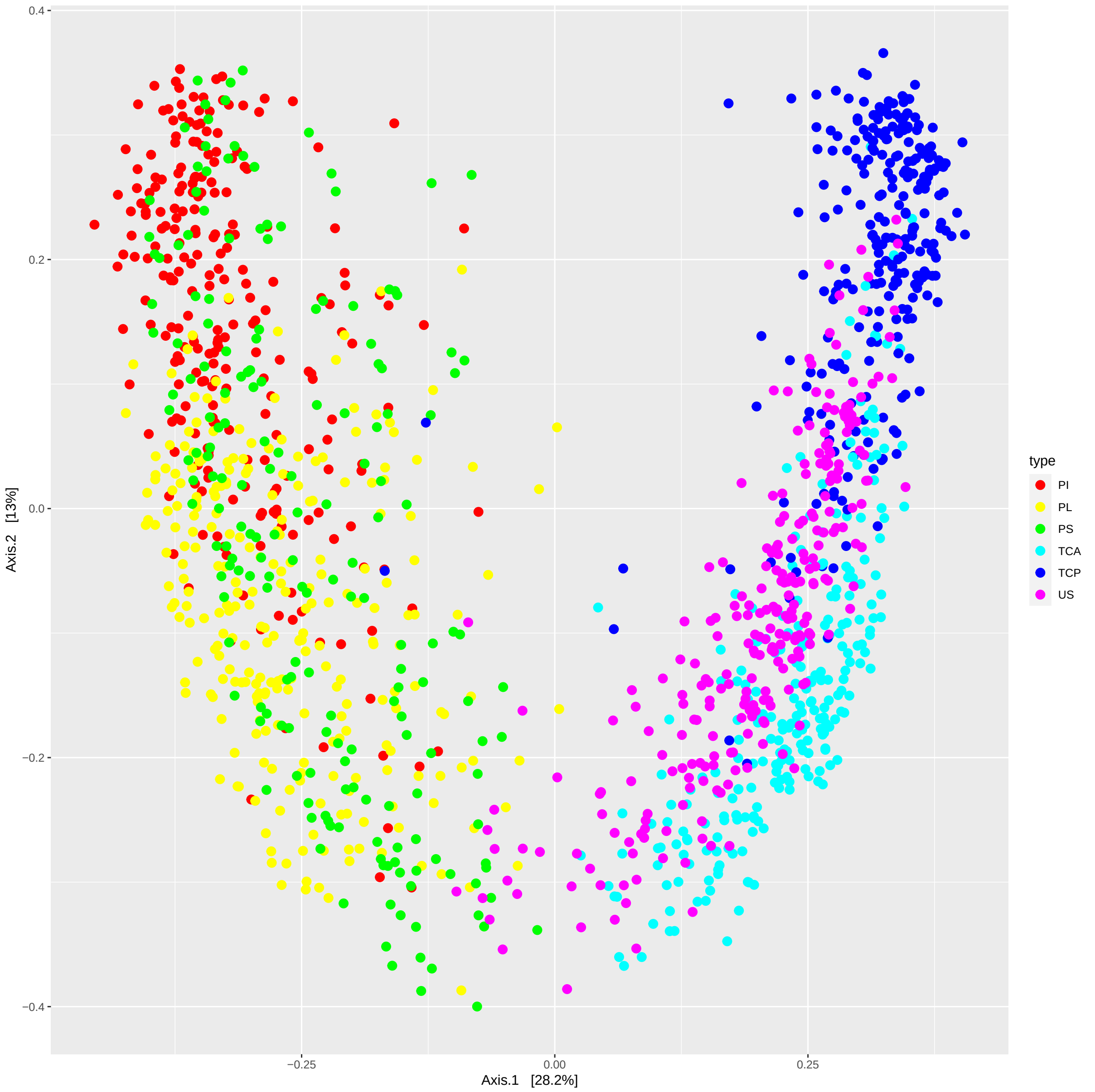


Figure S 7: **PCA plot of the doda microbiome.** The plaque and mucosal samples are split in the same way that Figure 2 of the main paper.

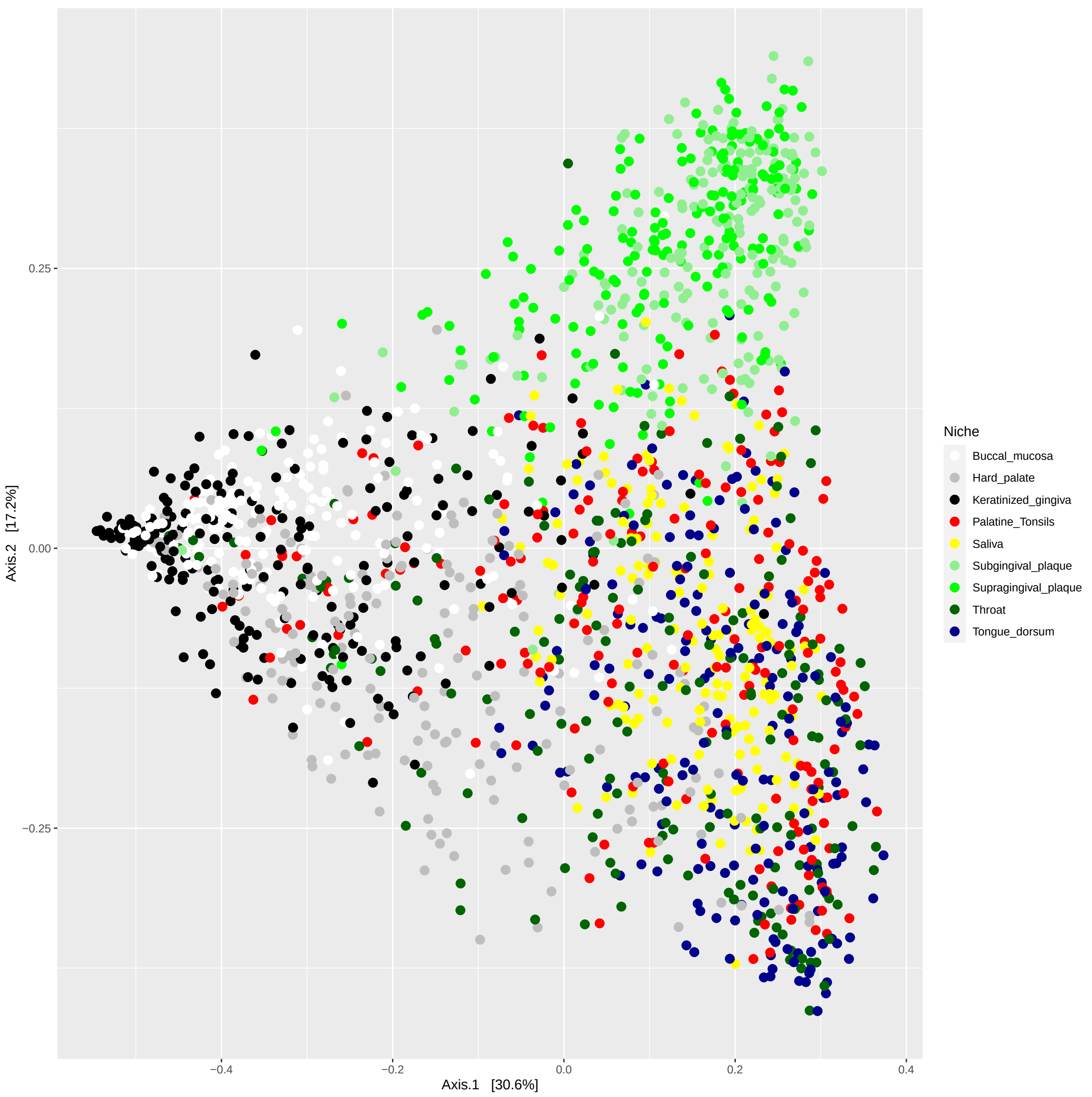


Figure S 8: **PCA plot of the oral niche of the HMP microbiome.**

# 4 Evaluation of the predictive model

This section gives more information on the predictive model we run (section 3.1). We aimed to predict the original niche (from the Dutch Oral Dataset) of the samples based on their sub-communities composition. This helps us to assess the meaningfulness of our sub-communities.

| Class | Sensitivity | Specificity | Pos Pred Value | Neg Pred Value | Precision | Recall | F1 | Prevalence | Detection Rate | Detection Prevalence | Balanced Accuracy |
|---|---|---|---|---|---|---|---|---|---|---|---|
| PI | 0.822 | 0.962 | 0.812 | 0.964 | 0.812 | 0.822 | 0.817 | 0.168 | 0.138 | 0.17 | 0.892 |
| PL | 0.849 | 0.939 | 0.747 | 0.967 | 0.747 | 0.849 | 0.795 | 0.175 | 0.149 | 0.199 | 0.894 |
| PS | 0.389 | 0.939 | 0.487 | 0.912 | 0.487 | 0.389 | 0.433 | 0.129 | 0.05 | 0.103 | 0.664 |
| TCA | 0.772 | 0.941 | 0.722 | 0.954 | 0.722 | 0.772 | 0.747 | 0.167 | 0.129 | 0.178 | 0.856 |
| TCP | 0.898 | 0.959 | 0.83 | 0.977 | 0.83 | 0.898 | 0.863 | 0.18 | 0.162 | 0.195 | 0.929 |
| US | 0.712 | 0.969 | 0.833 | 0.938 | 0.833 | 0.712 | 0.768 | 0.181 | 0.129 | 0.155 | 0.84 |

Multi-class area under the curve: 0.8566
F1 score: 0.8169014
Cohen's Kappa score: 0.7067146
Matthews correlation coefficient: 0.7076921
MultiLogLoss: 0.6524371

# 5 Other taxonomic structure

## 5.1 LDA Run with 12 sub-communities on DODA

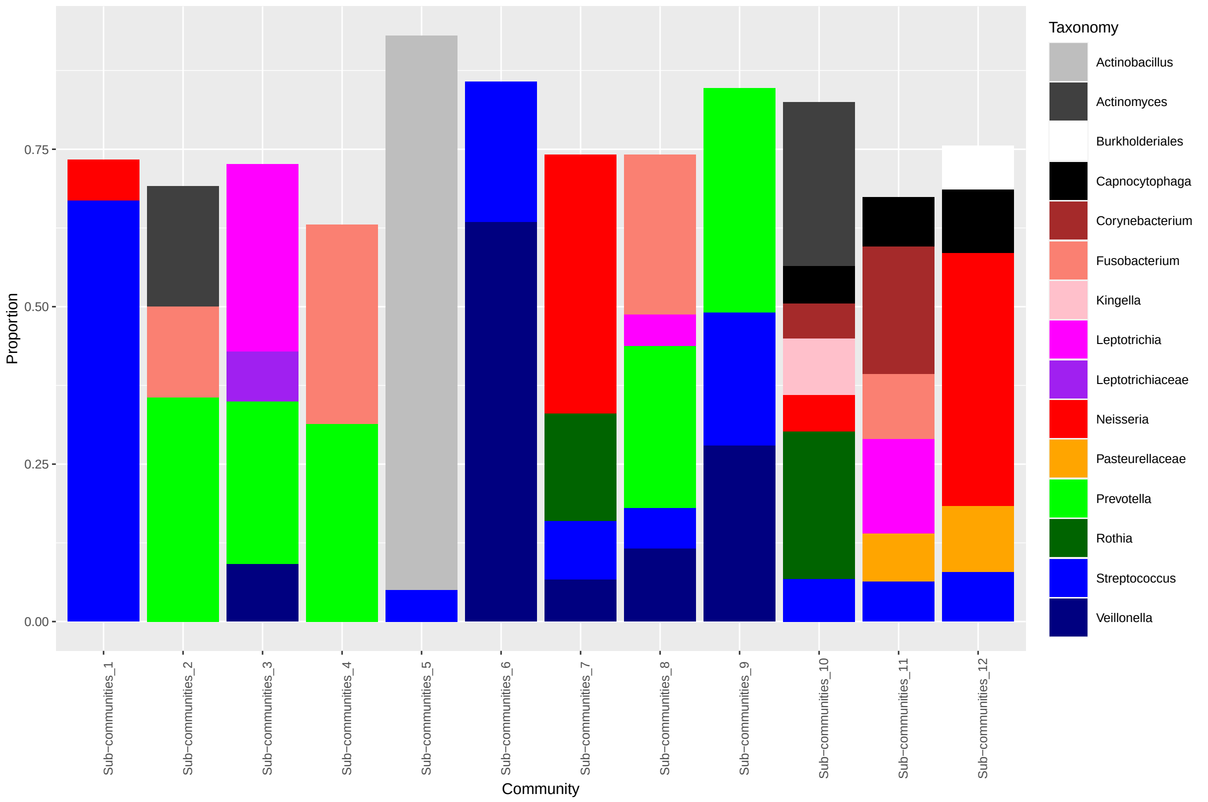


Figure S 9: **Composition of LDA-derived sub-communities using $K = 12$.** While the sub-communities have been built on OTU level, we plot here the lowest taxonomic level available for each OTUs. Only the taxa contributing for at least 5 % of a sub-communities are plotted here.

## 5.2 Taxonomic structure for HMP per niche

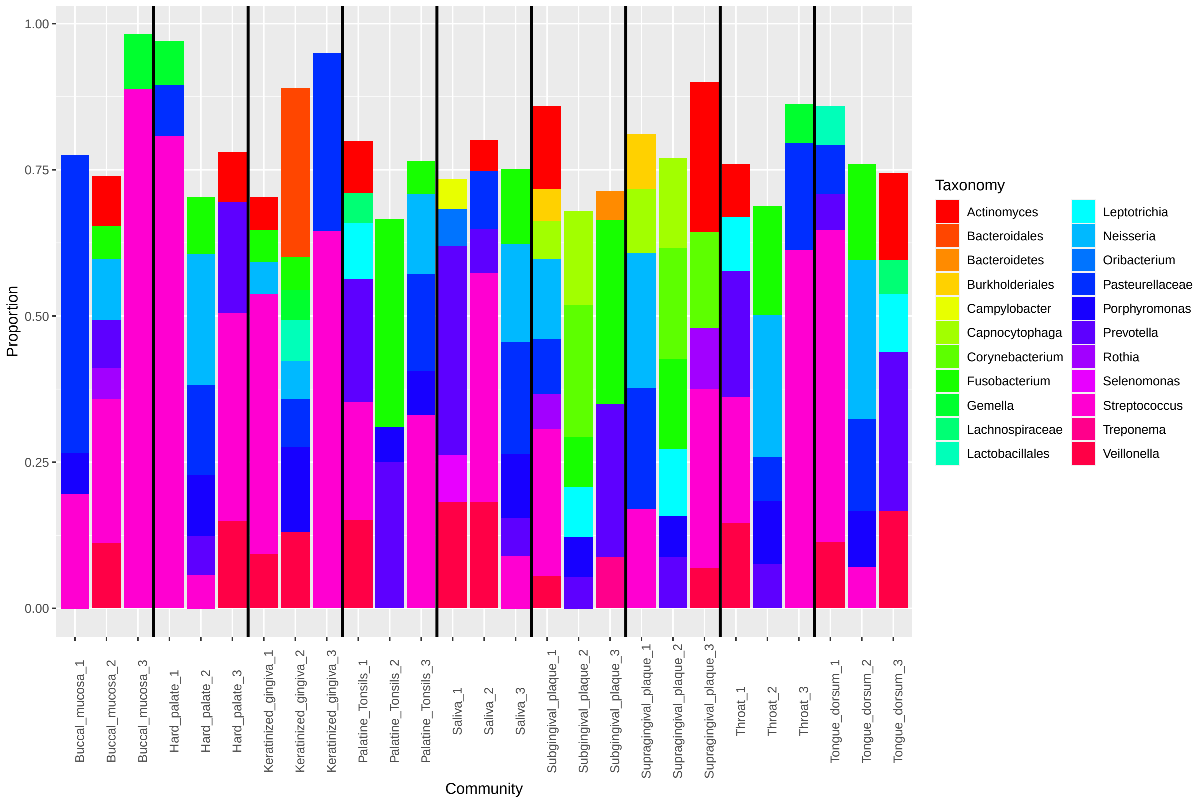


Figure S 10: **Composition of LDA-derived sub-communities using $K = 3$ for each oral niche from HMP dataset.** While the sub-communities have been built on OTU level, we plot here the lowest taxonomic level available for each OTUs. Only the taxa contributing for at least 5 % of a sub-communities are plotted here.

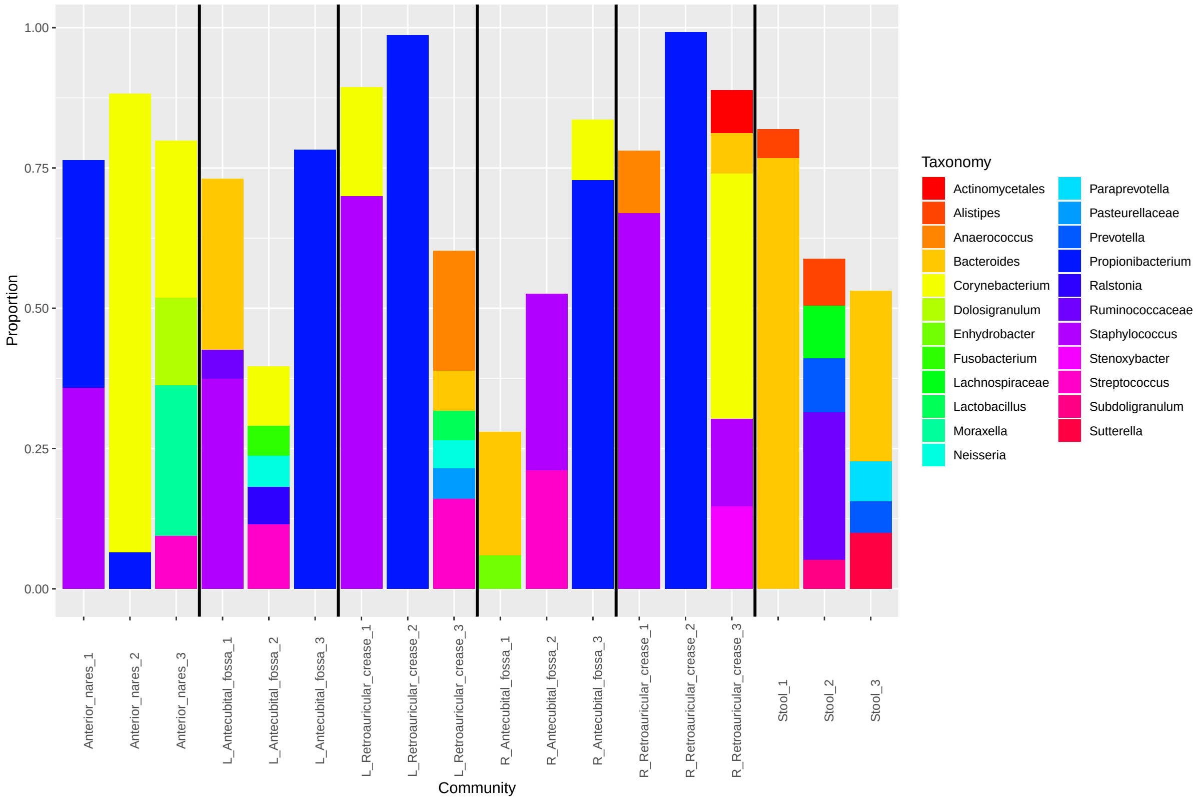


Figure S 11: **Composition of LDA-derived sub-communities using $K = 3$ for each skin and stool niche from HMP dataset.** While the sub-communities have been built on OTU level, we plot here the lowest taxonomic level available for each OTUs. Only the taxa contributing for at least 5 % of a sub-communities are plotted here.

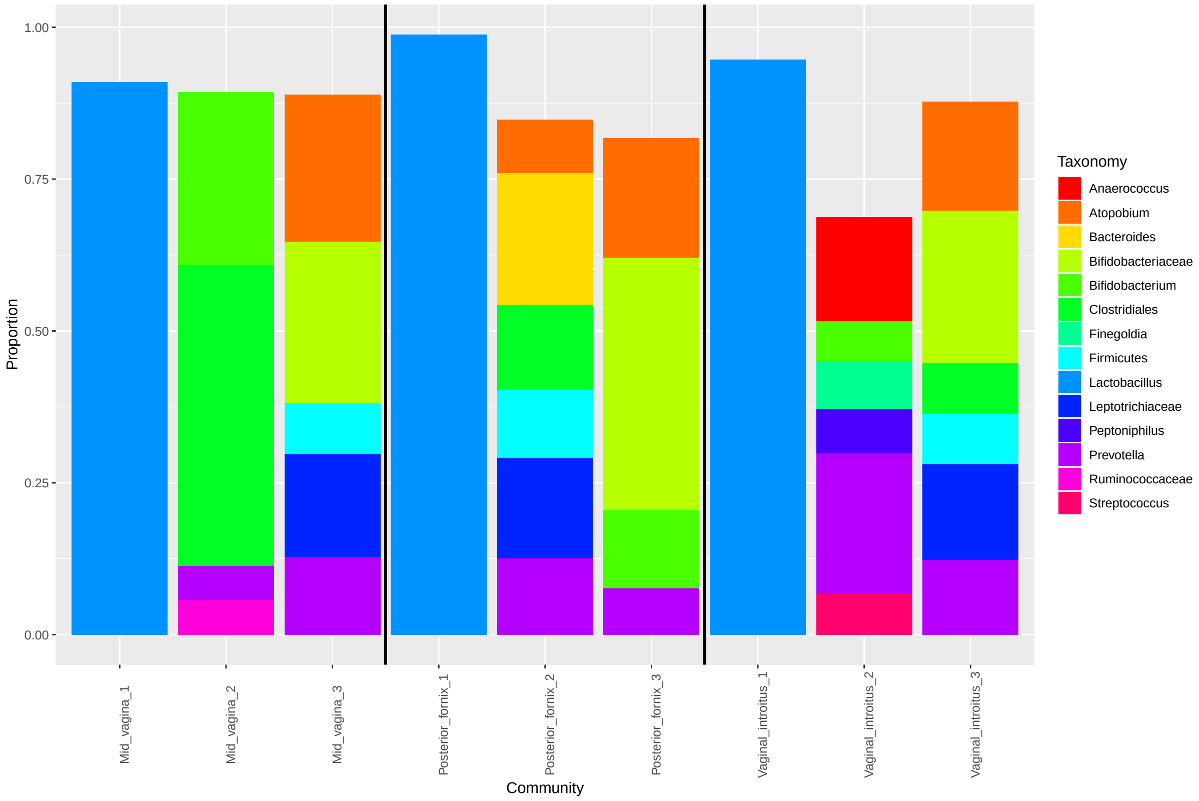


Figure S 12: **Composition of LDA-derived sub-communities using $K = 3$ for each vaginal niche from HMP dataset.** While the sub-communities have been built on OTU level, we plot here the lowest taxonomic level available for each OTUs. Only the taxa contributing for at least 5 % of a sub-communities are plotted here.

# 6 Insights about salivary sub-communities

In this section we propose to link our salivary sub-communities with a set of covariates. We performed spearman correlation test (for numerical) wilcoxon-test (for categorical) between the sub-communities proportion between sample and a set of covariates. This allow us to have a more detailed pictures than using raw OTUs since in our case a same OTU can have different biological interpretation du to the other species in the sub-community.

For example, we can see in Figure 13 even though the sub-communities 3 and 4 have similar taxonomic composition, we observe that sub-community 4 is significantly correlated with many covariates while sub-community 3 is not.

Those two sub-community are indeed composed of a *Prevotella-Streptococcus-Veillonella* combination but the prescence of other OTUs in the sub-community provided different results.

Finally, we can point out that the second sub-community is highly correlated with Lysozome and with a lower salivary pH.

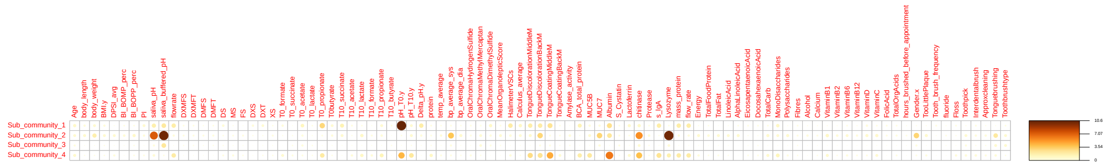


Figure S 13: **Heatmap of the -log10 of the p.values correlation between sub-communities proportion and other covariates.** Some sub-communities are highly correlated with clinical or chemical variables.

# 7 Simulation study and fixing the number of sub-communities

## 7.1 Simulation study

In this section, we present the output of our simulation study. We simulated a total of 20 different datasets using LDA generative process. The latent matrices we used as input were the ones obtained by applying the `LDA` function to our real dataset. Then, we applied the `dmn` function to check if the number of sub-communities proposed by `dmn` checked the true number of sub-communities used to generate our simulated datasets ($K = 5$). It happened that the number of estimated sub-communities ($K_{est}$) clearly depends of the number of included samples. The `dmn` function indeed overestimates the true number of sub-communities if the number of samples if too high. Therefore, we are confident that our choice to use a lower number of sub-communities than the one proposed by `dmn` when applying `LDA` to all niches simultaneously was relevant.

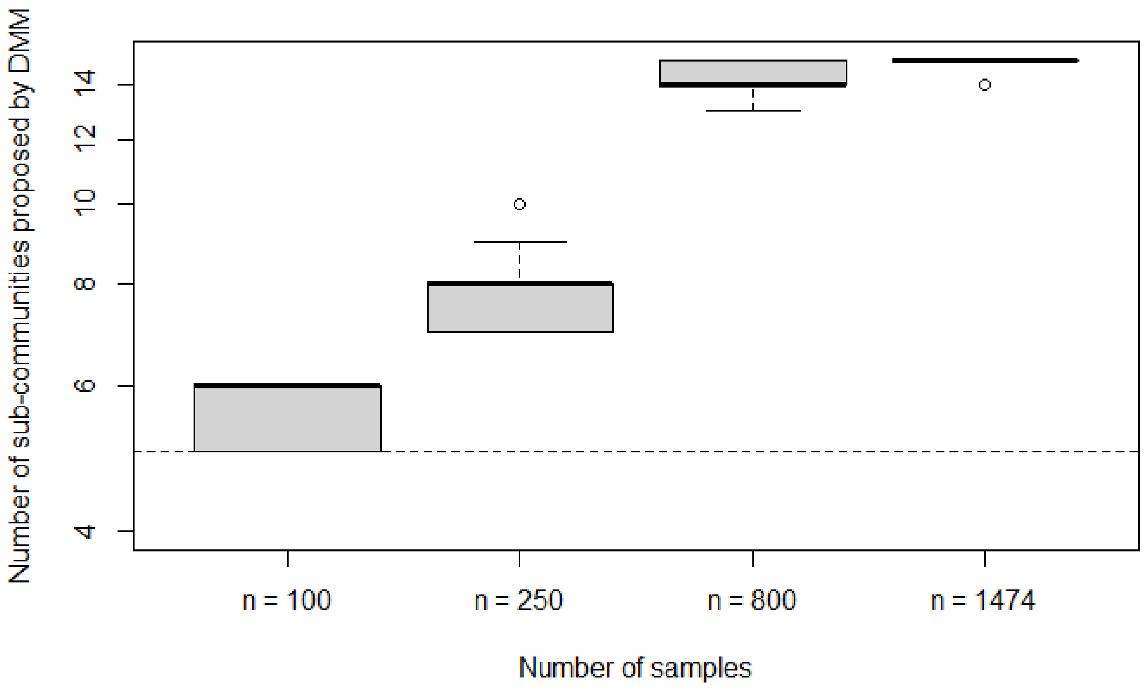


Figure S 14: **Number of sub-communities proposed by DMM on the LDA simulated dataset.** The true number of sub-communities corresponds to the dotted line.

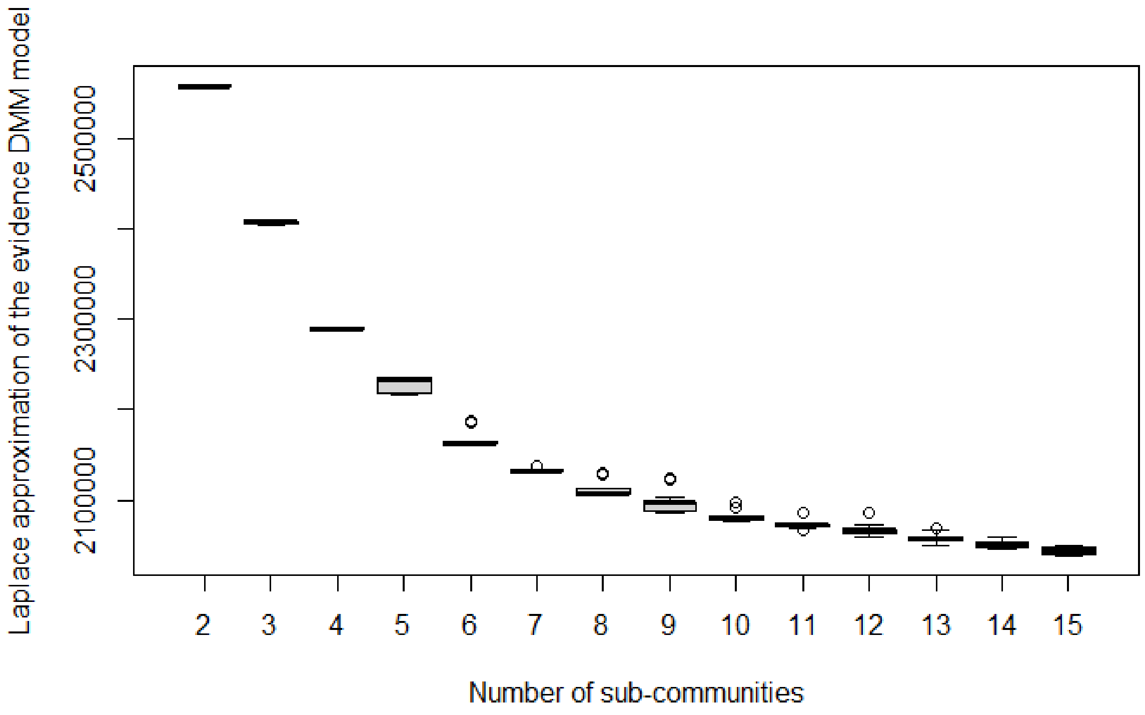


Figure S 15: **Index used by DMM to estimate the best number of sub-communities for $n = 1474$.**

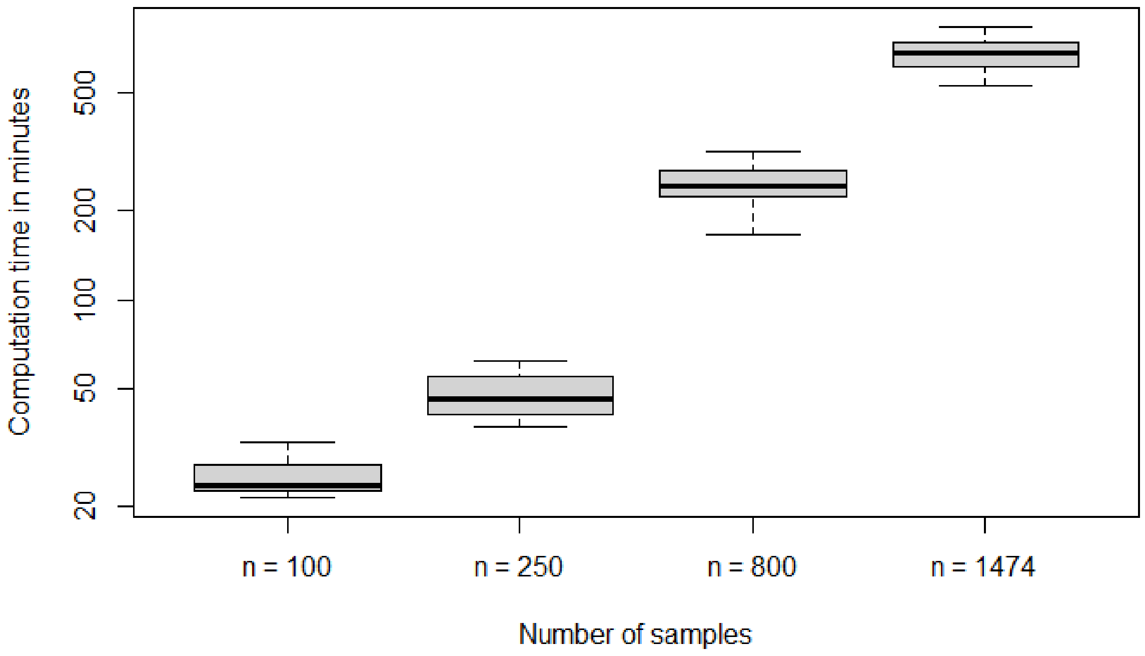


Figure S 16: **Computation time needed to fit the DMM model for $n$ varying from 100 to 1474.**

## 7.2 Usual metrics to fix the number of communities

We plot in this section the perplexity and the coherence-score based on the number of sub-communities for the complete DODA.

It appeared that those metrics did not help us to fix our number of sub-communities. On the one hand, The coherence score only varied marginally between $K = 2$ and $K = 12$. On the other hand, the

perplexity plot did not present outstanding elbows and it remained difficult to assess the best $K$ based on that plot.

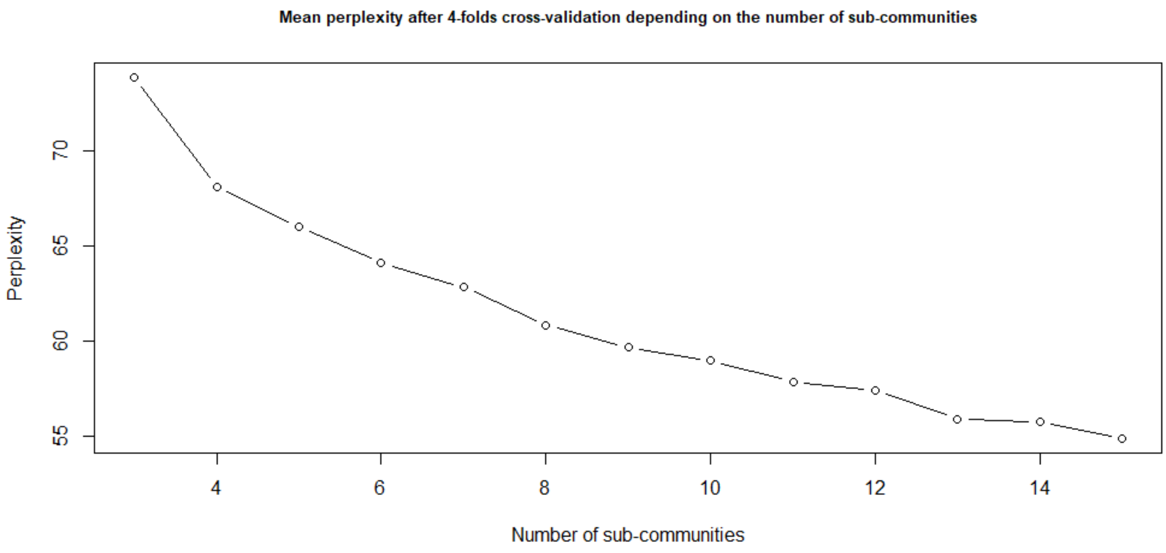


Figure S 17: Mean of the cross-validated perplexity with a 4-fold cross validation depending on the number of sub-communities.

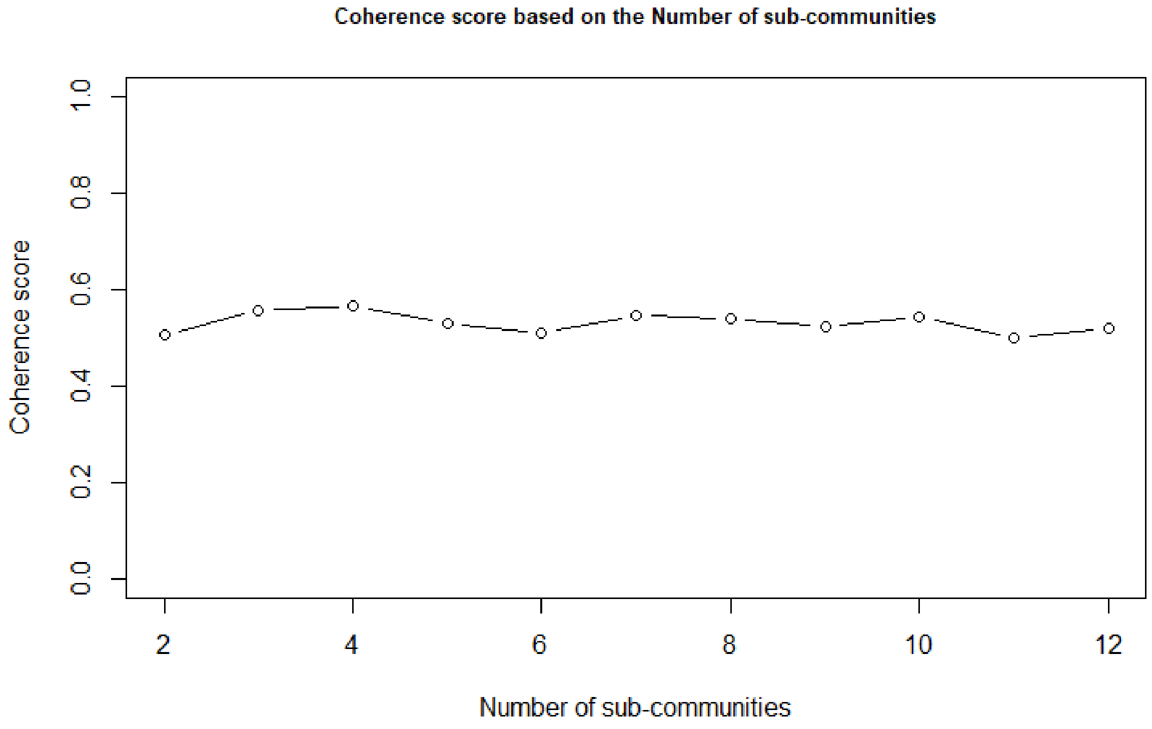


Figure S 18: Coherent score based on the number of sub-communities. We can see it remains stable regardless of $K$.

# 8 Similarity analysis with Bray-Curtis distance

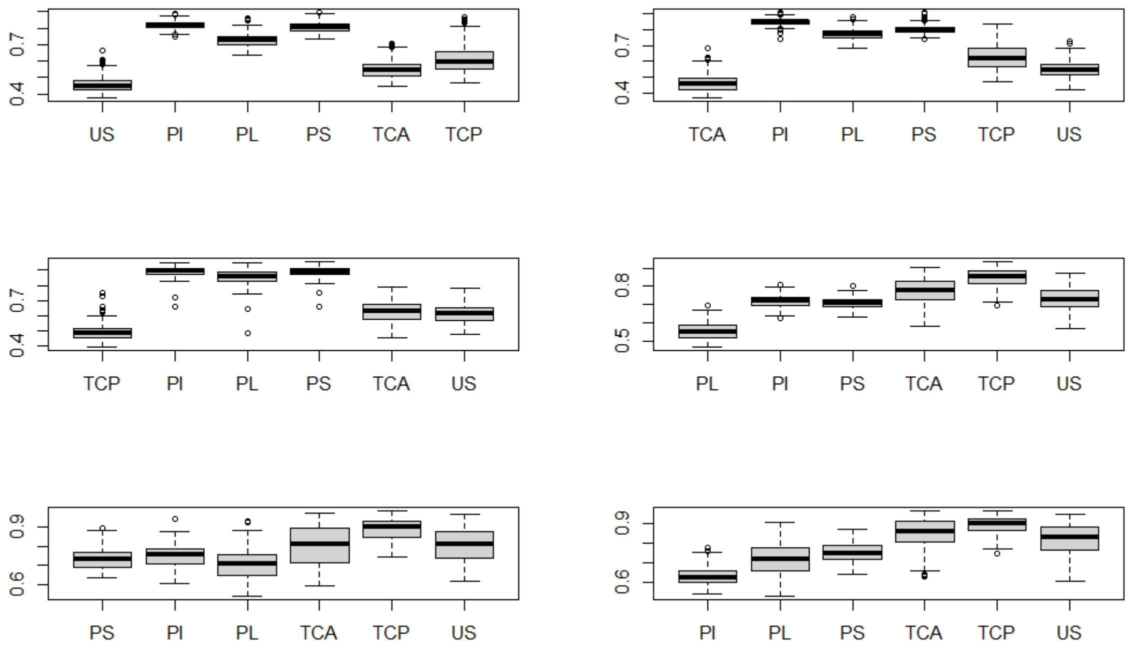


Figure S 19: Mean Bray-Curtis distance between the oral niches. The reference niche is the first one in each boxplot. Lecture: The median Bray-Curtis distance between sample coming from PI and US is 0.8. The unstimulated saliva is closer from TCA and TCP than the other niches.

# 9 LDAvis example output

Here we plot some output forn the *LDAvis* package which help us to choose the number of communities. We can see that $K = 5$ is the highest number of sub-communities such we dont observe overlapping in 2 dimensions visualization.

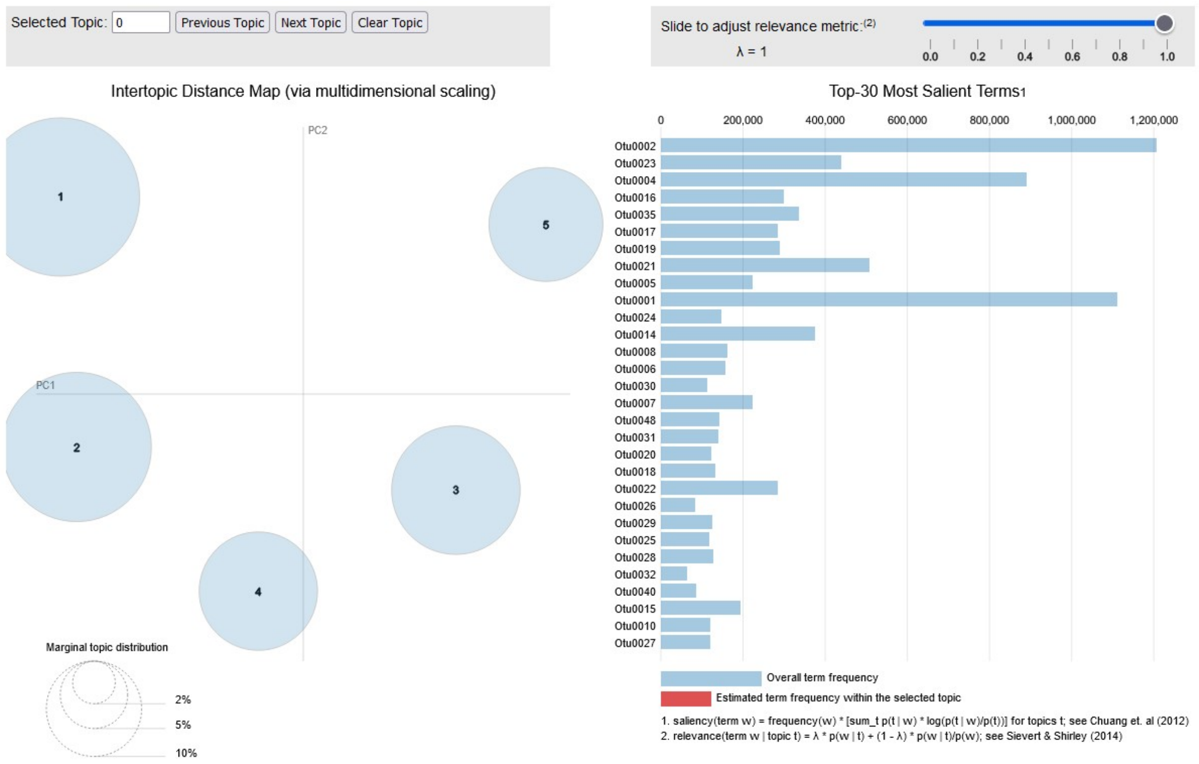


Figure S 20: Output of the LDAvis package for $K = 5$. We can see that there is no overlapping between our sub-communities.

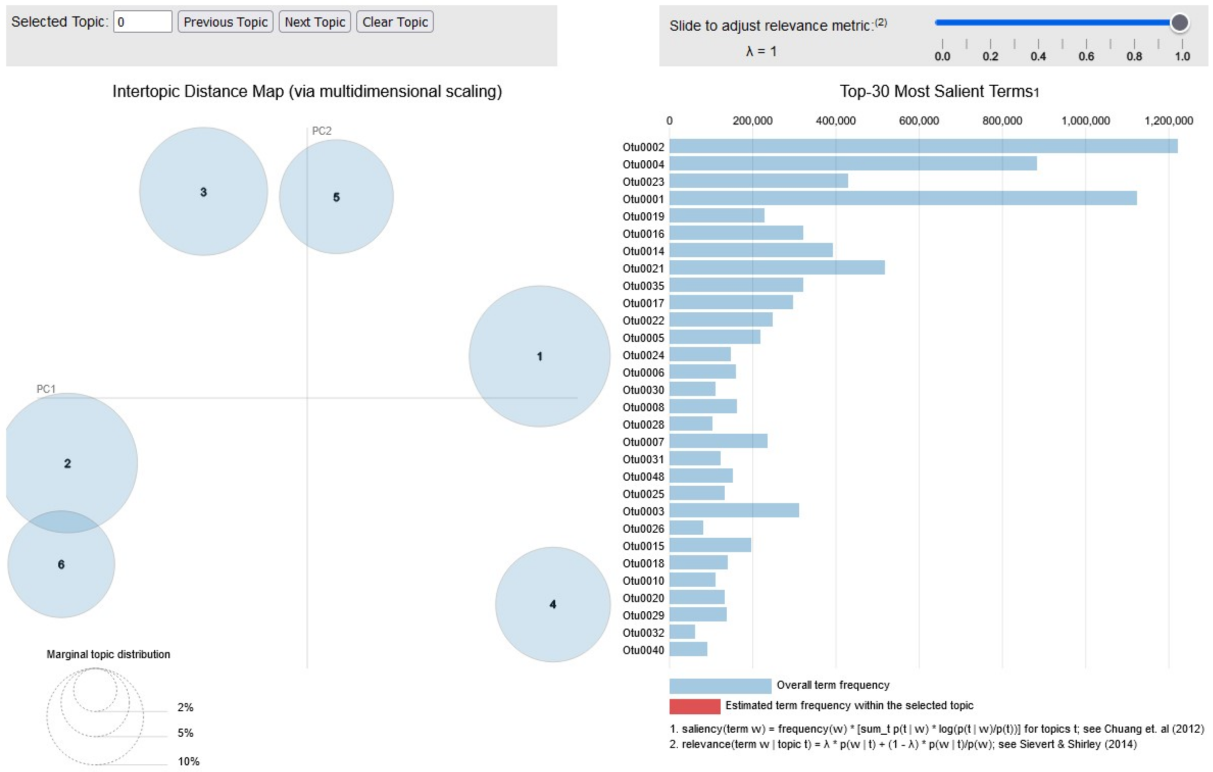


Figure S 21: Output of the LDAvis package for $K = 6$. Here, sub-communities 2 and 6 present similar composition.

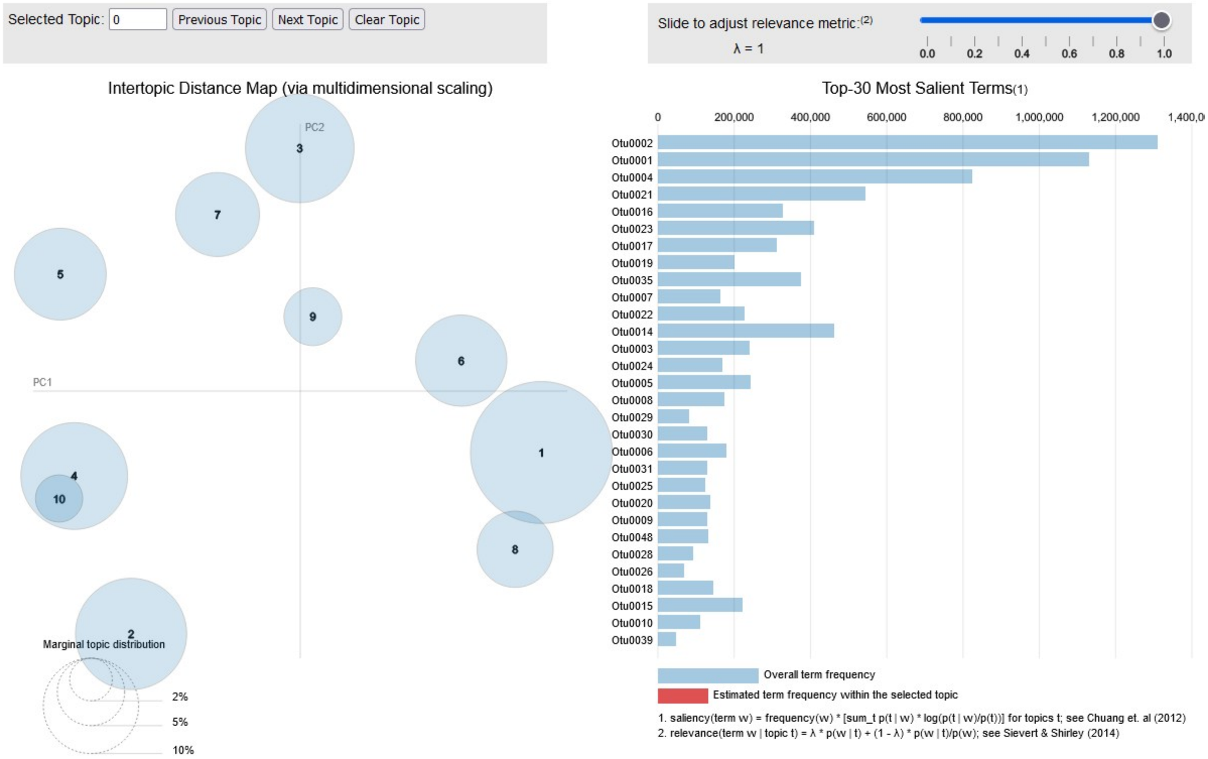


Figure S 22: Output of the LDAvis package for $K = 10$. While the number of sub-communities increases, we observe more overlapping and some sub-communities present very few reads (number 10).

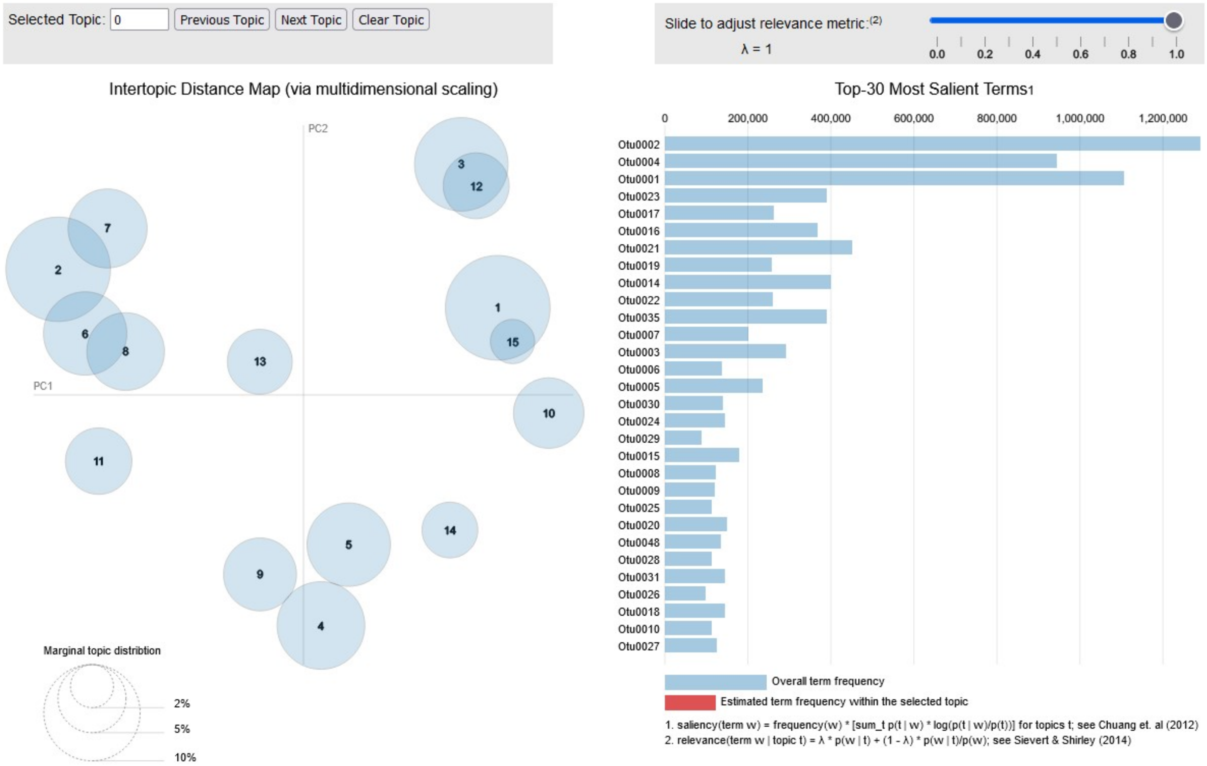


Figure S 23: Output of the LDAvis package for $K = 15$. As for $K = 10$, more and more sub-communities have very few reads or overlapped with each others.
